# Rationalizing the Binding Modes of PET Radiotracers Targeting the Norepinephrine Transporter

**DOI:** 10.3390/pharmaceutics15020690

**Published:** 2023-02-17

**Authors:** Anna Tutov, Xinyu Chen, Rudolf A. Werner, Saskia Mühlig, Thomas Zimmermann, Naoko Nose, Kazuhiro Koshino, Constantin Lapa, Michael Decker, Takahiro Higuchi

**Affiliations:** 1 Pharmaceutical and Medicinal Chemistry, Institute of Pharmacy and Food Chemistry, University of Würzburg, D-97074 Würzburg, Germany; 2 Nuclear Medicine, Faculty of Medicine, University of Augsburg, D-86156 Augsburg, Germany; 3 Department of Nuclear Medicine and Comprehensive Heart Failure Center, University Hospital Würzburg, D-97080 Würzburg, Germany; 4 Division of Nuclear Medicine, The Russell H Morgan Department of Radiology and Radiological Science, Johns Hopkins University School of Medicine, Baltimore, MD 21205, USA; 5 Faculty of Medicine, Dentistry and Pharmaceutical Sciences, Okayama University, Okayama 700-0082, Japan; 6 Department of Systems and Informatics, Hokkaido Information University, Ebetsu 069-0832, Japan

**Keywords:** positron emission tomography, norepinephrine transporter, sympathetic nervous system, structure–activity relationships, T-shaped π–π stacking

## Abstract

Purpose: A new PET radiotracer ^18^F-AF78 showing great potential for clinical application has been reported recently. It belongs to a new generation of phenethylguanidine-based norepinephrine transporter (NET)-targeting radiotracers. Although many efforts have been made to develop NET inhibitors as antidepressants, systemic investigations of the structure–activity relationships (SARs) of NET-targeting radiotracers have rarely been performed. Methods: Without changing the phenethylguanidine pharmacophore and 3-fluoropropyl moiety that is crucial for easy labeling, six new analogs of ^18^F-AF78 with different *meta*-substituents on the benzene-ring were synthesized and evaluated in a competitive cellular uptake assay and in in vivo animal experiments in rats. Computational modeling of these tracers was established to quantitatively rationalize the interaction between the radiotracers and NET. Results: Using non-radiolabeled reference compounds, a competitive cellular uptake assay showed a decrease in NET-transporting affinity from *meta*-fluorine to iodine (0.42 and 6.51 µM, respectively), with *meta*-OH being the least active (22.67 µM). Furthermore, in vivo animal studies with radioisotopes showed that heart-to-blood ratios agreed with the cellular experiments, with AF78(F) exhibiting the highest cardiac uptake. This result correlates positively with the electronegativity rather than the atomic radius of the *meta*-substituent. Computational modeling studies revealed a crucial influence of halogen substituents on the radiotracer–NET interaction, whereby a T-shaped π–π stacking interaction between the benzene-ring of the tracer and the amino acid residues surrounding the NET binding site made major contributions to the different affinities, in accordance with the pharmacological data. Conclusion: The SARs were characterized by in vitro and in vivo evaluation, and computational modeling quantitatively rationalized the interaction between radiotracers and the NET binding site. These findings pave the way for further evaluation in different species and underline the potential of AF78(F) for clinical application, e.g., cardiac innervation imaging or molecular imaging of neuroendocrine tumors.

## 1. Introduction

The norepinephrine transporter (NET), which controls neurotransmission by regulating the reuptake of the neurotransmitter norepinephrine (NE) in the synaptic cleft [1], is an important theranostic target in multiple areas, including the diagnosis of cardiac diseases by monitoring the function of the sympathetic nervous system (SNS), the diagnosis and treatment of neuroendocrine tumors, and the early detection of Parkinson’s disease (PD) [2,3,4]. Radionuclide-labeled tracers targeting the NET are NE analogs that mimic NE turnover via NET-mediated uptake and NE storage within the neuronal terminals; these compounds are exemplified by *meta*-iodobenzylguanidine (MIBG), which has been in use for over 30 years as a single-photon emission computed tomography (SPECT) diagnostic tracer and has recently been approved by the US Food and Drug Administration (FDA) as a therapeutic agent [5]. In addition, 1-(3-bromo-4-(3-[^18^F]fluoropropoxy)benzyl)-guanidine (FBBG), which was selected from several candidates with diverse pharmacophores [6], has completed a phase 1 clinical trial [7].

**Figure 1:**
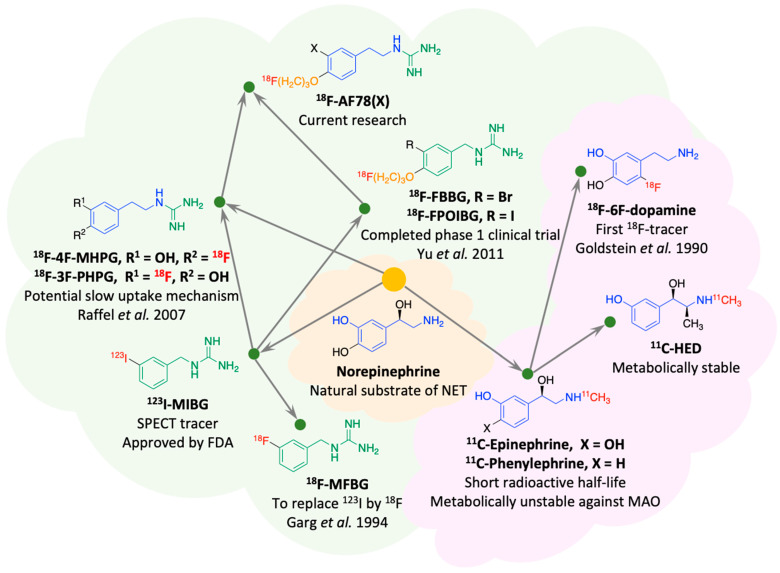
Evolutionary tree for the development of selected radiotracers targeting the norepinephrine transporter (NET), which can be categorized as monoamines (violet cloud) or guanidines (light green cloud) [4,8,9,10,11]. Blue and green represent the shared core structures of the radiotracers, orange represents the common “tail” structure, and red represents radionuclides.

Over the years, several NET radiotracers, especially those using positron emission tomography (PET) technology, have been developed, and these tracers can be divided into two categories according to their core chemical structure: primary or secondary amines, and metabolically stable guanidines (Figure 1) [4,8,9,10,11]. Among them, fluorine-18-labeled compounds have been a special focus of research in recent years due to their intrinsic advantages with PET technology and the longer radioactive half-life of fluorine-18 compared with carbon-11 (110 min vs. 20 min) [12]. Some researchers have focused more on benzyl guanidine derivatives, the pharmacophore of MIBG [4,6,13], while others have developed phenethylguanidine-based analogs [10]. On the one hand, among the candidate tracers reported, some have already proven their general feasibility in human studies [14,15,16]. On the other hand, several drawbacks, including blood flow dependency, low heart-to-liver ratios or challenging radiolabeling protocols with poor labeling yields, have indicated room for improvement. To counteract some of these drawbacks, we recently reported a new NET-targeting radiotracer, AF78, based on a phenethylguanidine core structure with efficient one-step labeling, which also demonstrated high NET affinity and advantageous in vivo kinetics across various species [17,18]. Nevertheless, from a structural point of view, little insight has been obtained for further optimization, even after information has been gathered on the certain similarities and differences between AF78 and other radiotracers (Figure 1). More importantly, in general, the development of radiotracers does not follow the principle of rational drug design that dominates drug discovery and development as the synthesis and characterization of new tracers are in fact fairly unsystematic with poor considerations or systematic investigations of the structure–activity relationships (SARs). This is due to several reasons. First, the complexity of developing new radiotracers requires the involvement of a multidisciplinary team, the availability of research facilities with a constant supply of radionuclides is limited, and companies are insufficiently involved. These limitations, however, did not hinder us in searching for new radiotracers that are more favorable for clinicians and more suitable for clinical application.

Based on our previously reported NET-targeting radiotracer AF78, by retaining the phenethylguanidine core structure for NET affinity as well as the 3-fluoropropyl moiety for convenient radiolabeling, we mainly focused on the *meta*-substitution using nonpolar *meta* halogen and polar hydroxyl derivatives. Through investigating this series of radiotracers, we intended to take the first step in exploring the sophisticated SARs of radiotracers targeting the NET by applying certain principles of rational drug design to the investigation of novel radiotracers.

## 2. Materials and Methods

### 2.1. Synthesis and Radiolabeling

A series of AF78(F) derivatives with *meta*-chlorine, bromine, iodine hydrogen and hydroxy was synthesized individually (Table 1) with the detailed experimental procedures and structural characterization shown in the Supplementary Material. The cold reference compounds (nonradioactive with fluorine-19) were used for competitive cellular uptake studies. The precursors for radiolabeling (with a leaving group to allow for displacement by fluorine-18) with different protecting groups on the guanidine moiety (*N,N′,N″*-tri-Boc protected or non-protected guanidine) as well as different leaving groups (tosylate or brosylate) were used in the radiolabeling procedure for the biodistribution and imaging studies. Radiolabeling was performed analogously according to a previously described protocol [17,18] with variations in terms of a different base and concentration of acid used in deprotection. Radiochemical yield (RCY) is calculated from the isolated final product after HPLC and cartridge purification deducted by radioactivity loaded onto the QMA cartridge with decay correction. A detailed experimental description can be found in the Supplementary Material.

### 2.2. Competitive Cellular Uptake Studies

Evaluation of all synthesized and reference compounds against the uptake of [^3^H]NE was performed in the human neuroblastoma cell line SK-N-SH stably expressing NET according to a previously described protocol using the NET selective inhibitor desipramine as a control and NE and MIBG as standard references [18]. A detailed experimental description can be found in the Supplementary Material. It should be noted that the NET affinities of the tested compounds are expressed as IC_50_ in the current manuscript instead of the dissociation constant *K*_d_. First, because all the tested compounds are substrates of NET that can be transported, they share the same binding site. The equation for competitive inhibitors in a Cheng–Prusoff relationship applies to the current situation: IC_50_ = *K*_d_(1 + [S]/*K*_m_), where [S] represents the substrate concentration, i.e., [^3^H]NE, and *K*_m_ represents the Michaelis constant of the substrate [23]. Second, the [S] calculated from the addition of 600 Bq ^3^H[NE] (1024.9 GBq/mmol, PerkinElmer LAS GmbH, Rodgau, Germany) is 1.17 nM, and the NE-NET Michaelis constant Km is 0.28 ± 0.03 µM [24], which gives the correction from Cheng–Prusoff equation as only 0.4%. Therefore, the IC_50_ value in the current study is considered equal to *K*_d_ without additional correction.

### 2.3. Animal Handling

The experimental protocols were approved by the Animal Ethics Committee of the National Cerebral and Cardiovascular Center, Research Institute, Osaka, Japan (approval number 18019) and conducted in strict accordance with the Guide for the Care and Use of Laboratory Animals published by the US National Institutes of Health [25] and the ARRIVE guidelines. Anesthesia was induced in male Wistar rats (n = 3–6 for each radiotracer, weighing 200–250 g on average, Charles River Laboratories, Research Models and Services, Germany GmbH, Sulzfeld, Germany) by using 5% isoflurane and maintained during the whole experiment with 2% isoflurane. Large animal PET imaging was performed on rhesus macaques (n = 2, weighing 3.9–4.2 kg, Primate Research Institute Kyoto University, Aichi, Japan). The induction of anesthesia in non-human primates (NHPs) was conducted by intramuscular injection of ketamine (1.5 mg/kg) and xylazine (0.6 mg/kg) to allow animal preparation and handling. After a tracheal cannula was inserted, 1.5% sevoflurane (SEVOFLURANE Inhalation Solution, Pfizer Japan Inc., Tokyo, Japan) vaporized with 100% oxygen was inhaled, and the tidal volume and respiratory rate of the ventilator were monitored and maintained in the normal range throughout the imaging sessions with an anesthesia workstation (Apollo^®^, Drägerwerk AG & Co. KGaA, Lübeck, Germany) [26].

### 2.4. PET Imaging and Biodistribution Study

Preliminary PET imaging of [^18^F]AF78(F) in rodents (n = 2) and NHPs (n = 2) was obtained according to a previously described protocol [18]. For the rat biodistribution studies (n = 3–6 for each tracer), radiotracers (1–2 MBq) were administered via the tail vein. Ten minutes after radiotracer administration, the animals were euthanized. The organs of interest were harvested for tissue counting with a γ-counter (2480 Automatic Gamma Counter WIZARD^2^, PerkinElmer LAS GmbH, Rodgau, Germany). Following weight and decay correction of the tissue counts, the heart-to-blood (H/B) ratios were calculated. A detailed experimental description can be found in the Supplementary Material.

### 2.5. Computational Modeling

Ligand structures were built in Molecular Operating Environment (MOE, version 2021.11, Chemical Computing Group, Montreal, QC, Canada) using MMFF94x as a forcefield and a root-mean-square (RMS) gradient of 0.001 kcal/mol∗Å [27]. The X-ray structure of a *Drosophila melanogasta* dopamine transporter with NET-like mutations (dDATNET) in L-norepinephrine bound form found by Pidathala et al. with a resolution of 2.88 Å was prepared in MOE as well by removing all non-protein molecules besides two sodium ions at the active site [28]. The resulting transporter was protonated to the pH, the crystal structure was obtained at pH = 7. All possible binding positions were generated using Genetic Optimization for Ligand Docking (version v2021.1.0, CCDC Software, Cambridge, UK) [29]. Fifty independent algorithm runs were carried out using the empirical scoring function GoldScore. Atom number 290 of the dDATNET, which represents one of the oxygen atoms of the acid function of ASP-46, was chosen for the flood fill atom with a flood fill radius of 15 Å. For further evaluation of the generated docking results, the given docking poses were re-scored using the knowledge-based scoring function DSX [30] (DrugScore eXtended) with the Cambridge Structural Database and the results were assigned to clusters using an RMS deviation of 2.0 Å as the cut off. The generated poses showed high convergence with 46 of 50 poses for AF78(F) assigned to the same cluster. The pose with the highest GoldScore was displayed in Pymol Molecular Graphics System (PyMOL, version 2.4.1, Schrödinger, LLC, New York, NY, USA).

## 3. Results

### 3.1. Synthesis and Radiolabeling

**Figure 2:**
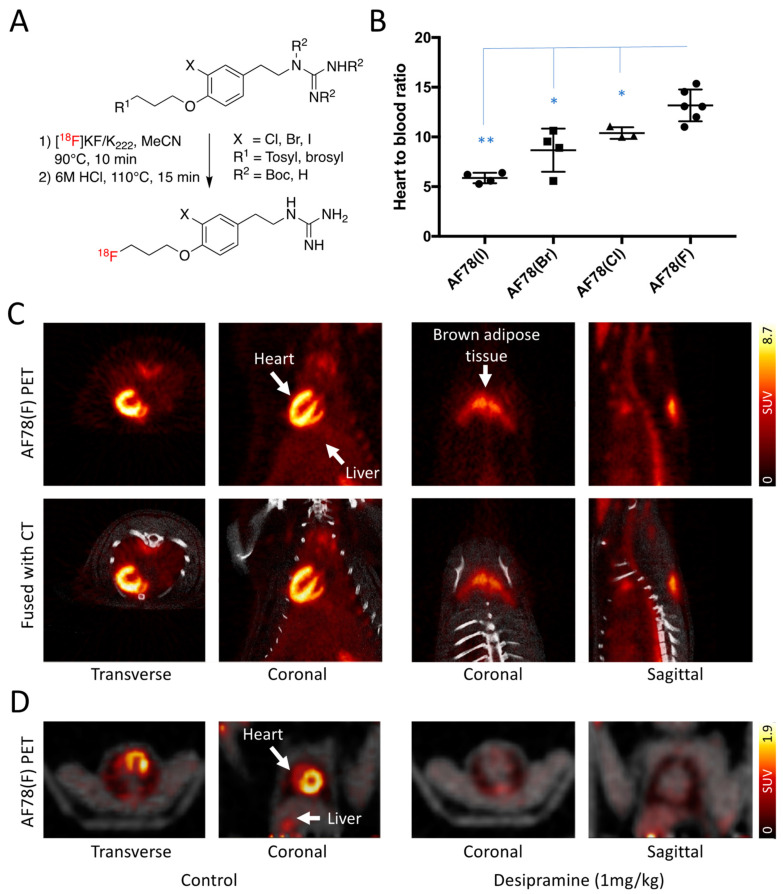
(**A**) Procedure for radiolabeling NET-targeting radiotracers using a one-pot two-step protocol where a radiofluorination was followed by the removal of Boc protecting groups under acidic conditions. (**B**) In vivo biodistribution experiments using radiolabeled tracers demonstrated that the heart-to-blood ratios agree well with the competitive cellular uptake assay, with AF78(F) showing the highest cardiac uptake. * *p* ≤ 0.05; ** *p* ≤ 0.01. (**C**) Fused PET/CT images of the cardiac (**left**) and brown adipose tissue (**right**) uptake of [^18^F]AF78(F) in healthy rats. (**D**) PET images (fused with transmission scan) with and without NET blockade with desipramine (1 mg/kg, i.v., 10 min before radiotracer injection) in non-human primates (NHPs). Homogeneous cardiac [^18^F]AF78(F) uptake can be reversed by pretreatment with desipramine. Cardiac imaging in rats and NHPs are generated from unpublished data using similar protocol as published previously. For further detailed kinetics in NHPs, please refer to [18].

Different cold references and precursors of the *meta*-substituted AF78 analogs were synthesized (Supplemental Figures S1–S6, Table 1, Figure 2A). In addition, instead of using a fully protected guanidine moiety with a triazole structure as previously reported to radiolabel [^18^F]AF78(F) [17], we also investigated how the structures of the precursors influence the RCY. The RCY varied depending on the structure of the precursor: (1) 27.9 ± 3.1% (n = 6) of AF78(F) with fully protected guanidine precursor; (2) 16 ± 3.5% (n = 3) and 9.5 ± 2.8% (n = 3) for AF78(I) and AF78(Br), respectively, with *N,N′,N″*-tri-Boc protected guanidine precursor; and (3) the RCY dramatically decreased for AF78(Cl) to 0.5 ± 0.1% (n = 3) while using a precursor with tri-Boc protected guanidine under similar radiolabeling protocol. The lack of full protection of the guanidine moiety, i.e., tri-Boc instead of di-Boc-triazinanone of AF78(F), in the precursors of the analogues led to this decrease. However, while using a precursor with non-protected guanidine, the RCY recovered to 10 ± 1.0%, and the general manual radiolabeling procedure could also be shortened from 120 min to 90 min (Supplemental Table S1, all results are decay-corrected based on the starting radioactivity loaded onto the QMA cartridge). The specific activities of all the target radiotracers were >390 GBq/mmol, and their radiochemical purities were >95%, as confirmed by analytical high-performance liquid chromatography (Supplemental Figures S7–S9).

### 3.2. Competitive Cellular Uptake Studies

The results show positive correlations between the tracers’ NET affinity and the physical properties of the *meta*-substituents with AF78(F) being the most potent, as previously reported [17], whereas AF78(I) was the least potent compound (Table 1, Supplemental Figure S10). From fluorine to iodine, the electron-withdrawing effect/electronegativity decreased from 3.98 to 2.66 according to the Pauling scale [19], and the atomic radius increased from 0.57 to 1.39 Å [20]. The IC_50_ values decreased concomitantly and showed significant differences compared with NE. AF78(OH) displayed a great loss in NET affinity, which was apparently due to the polar *meta*-hydroxy substituent in contrast to the nonpolar halogen atoms in the *meta* position of the other analogs. In contrast, the affinities are not correlated with the atomic radius or the volume of the substituents (Table 1). 

### 3.3. Biodistribution and PET Imaging

The H/B ratios of the halogenic AF78 analogs, calculated from a biodistribution study in rats measured 10-min after the administration of fluorine-18-labeled tracers, revealed a trend of decreasing cardiac uptake when the halogen substituent moved from the top to the bottom of the periodic table column, which was in good agreement with their NET affinities obtained from the cellular studies. Higher cardiac uptake (expressed as heart-to-blood ratios) was observed for the compounds containing halogens with smaller atomic radii and electronegativities, such as fluorine (13.2 ± 1.2) and chlorine (10.4 ± 0.4), compared with the compounds with the larger halogens bromine (8.7 ± 1.4) and iodine (5.9 ± 0.4) (Figure 2B). All the newly prepared analogues of AF78(F) showed significant difference in H/B ratios (*t*-test using GraphPad Prism 8.4.3, GraphPad Software, Boston, MA, USA).

PET/computed tomography (CT) imaging with [^18^F]AF78(F) was performed using a small animal PET imaging protocol, applied as described previously [17,18]. Systemic distribution of the radiotracer in healthy rats showed homogenous distribution throughout the left ventricular wall. Delineation of the right ventricular wall was also visible due to the partial volume effect caused by the thinness of the right ventricular wall (Figure 2C, left). Specific uptake via the NET was proven by uptake in brown adipose tissue since thermogenesis is primarily driven by the SNS (Figure 2C, right). Therefore, this tracer also has the potential to monitor SNS activity in brown adipose tissue in rat models of diabetes and obesity [31] or in clinical applications [32]. PET imaging in nonhuman primates (NHPs) demonstrated distinct NET-mediated uptake in the heart with specificity proven by pretreatment with the selective NET inhibitor desipramine (1 mg/kg, i.v.) 10 min before tracer injection (Figure 2D).

### 3.4. Computational Studies

**Figure 3:**
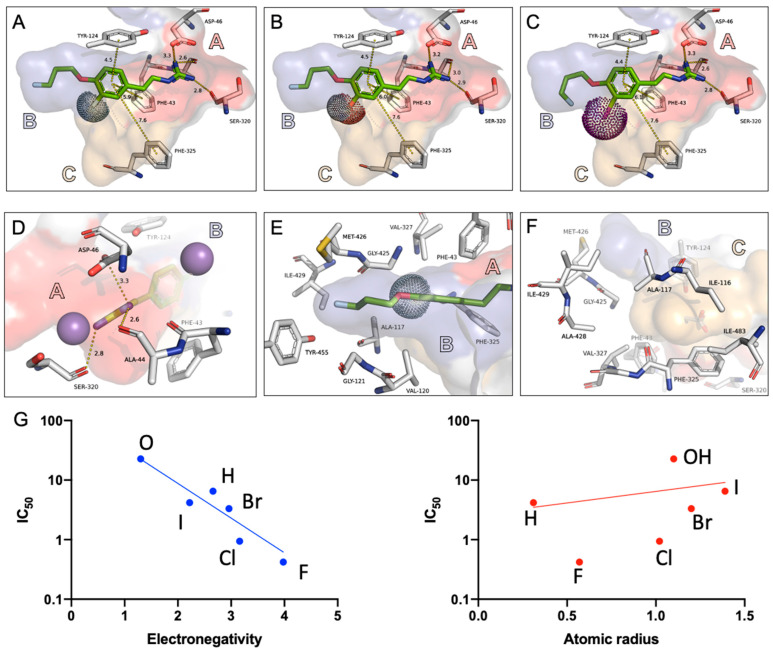
Different substitution pattern of the AF78 series (backbone of the common structure in green) in complex with *Drosophila* dopamine transporter with NET-like mutations (dDATNET, PDB6M0Z) [23]. The space occupied by the halogen atoms or the phenol function are visualized by dots. The two sodium ions of the transporter are shown as purple spheres out of scale for a better overview. The binding pockets are designated with letters A–C. Distances between the presented tracer and the transporter are shown in Å. The atomic radius of the halogens and the hydroxy group are displayed with dots in A–C. (**A**–**C**) Proposed binding mode of AF78(F), (OH) and (I), respectively, bound to the dDATNET. (**D**) Pocket A interactions of the guanidine moiety with ALA-44, ASP-46, SER-320 and two sodium ions (displayed in purple). (**E**) Visualization of Pocket B from a top-down perspective, which is surrounded by a group of nonpolar amino acid moieties (GLY-121, VAL-120, MET-426, ILE-429). (**F**) Amino acids forming the hinge region between Pocket B and C (ALA-117, GLY-425) and the surrounding nonpolar amino acid moieties that form the lipophilic Pocket C (ILE-113, ILE-483, VAL-327, ALA-117, VAL-113, PHE-325). (**G**) Correlation between NET affinities of cold references obtained from competitive cellular uptake assay and physical properties of the substituents. The IC_50_ values of the target tracer correlated positively with the electronegativity instead of the size of the meta-substituent on the benzene-ring.

The crystal structure of a dopamine transporter (PDB: 6M0Z) with human NET (*h*NET)-like mutations was used as a surrogate of NET for the exploration of the binding modes of the listed compounds via a molecular docking approach [29]. The 3.3 Å resolution of the crystal structure hardly allows distinct interpretation of interactions at the transporter. Additionally, the dopamine transporter used by Pidathala et al. originates from *D. melanogasta*. In this study, however, the discussed crystal structure was solely applied to a surrogate docking model for indications of binding parameters. In this structure, three binding pockets were assigned to predict the binding poses and affinities of the NET inhibitors (Figure 3A–F). Pocket A, formed by sodium cations and several polar amino acid residues, is important for the interaction with the positively charged amino or guanidine groups of NET-targeting molecules. Pocket B, which is formed by a group of nonpolar amino acids into a tube shape, is perfectly suitable for an interaction of long aliphatic chains (Figure 3E). Pocket C constitutes a highly lipophilic environment as well, as seen by the amino acids building these surroundings (Figure 3F). The hinge between the latter two pockets is triggered by ALA-117 and GLY-425, which both lack a suitable motif for a hydrophilic interaction (Figure 3F).

The docking experiments suggest a binding of the AF78 series of radiotracers to the primary binding site of the dDATNET with the guanidine moiety pointing into pocket A (Figure 3A–C). The 3-fluoropropyl moiety fits into the lipophilic pocket B mimicking one of the aromatic rings present in related antidepressants. The benzene ring is well surrounded by various aromatic amino acids, such as TYR-124, PHE-325 and PHE-43. The space available at the pockets is very limited by the fact that the phenylethylamine scaffold already reaches far back into pocket B (Figure 3A–C). The binding affinity may therefore be influenced not only by the electron-withdrawing effects of the substituent on the benzene ring but also by the size of the substituent in *meta*-position. The radius of the halogen in AF78(I) already causes a clash with the surface of the investigated transporter. In the series of increasing radii from F > Cl > Br > I, the affinity to the target decreases. For AF78(F), the fluorine atom decreases the electron density of the benzene ring while forming a partially positively charged hydrogen atom that points at the negatively charged π cloud of the benzene ring of the tyrosine moiety. As stated earlier, these conclusions have to be taken tentatively considering the applied surrogate model. 

## 4. Discussion

By replacing the *meta*-fluorine of the novel NET radiotracer AF78(F) with either electron-withdrawing or electron-donating groups—halogens, a hydrogen or a hydroxyl group—we systemically investigated the SARs of NET-targeting tracers and clarified the rationale for the tracer-NET interaction. For this purpose, a common synthetic route applying a Sandmeyer reaction was successfully developed, which allowed late-stage derivatization by sharing a common intermediate to conveniently and economically prepare a series of analogs. Due to overall low yields for preparation of AF78(OH) applying the Sandmeyer reaction compared with AF78(Cl) and AF78(Br), we chose to start from vanillin in this case, which naturally contains oxygens in the para and meta positions. In addition, partial dehalogenation took place during the preparation of AF78(Cl) and (Br), which prompted us to also include the defunctionalized AF78(H) in the testing. When using precursors with *N,N′,N″*-tri-Boc protected guanidine, the RCYs varied greatly and were inconsistent with the physicochemical properties of the *meta*-substituents. No explanation could be given to clarify why *meta*-chloride had the lowest RCY. Surprisingly, it recovered a little to 10 ± 1.0% when using a precursor with non-protected guanidine. The resonance structures of non-protected guanidine may represent low nucleophilicity than *N,N′,N″*-tri-Boc protected ones with one NH that can be extremely easy deprotonated in the basic radiofluorination condition, and therefore, influence the fluorination yield drastically. A further investigation using precursors with non-protected guanidine will be continued. The initial rationale for introducing the 3-fluoropropyl moiety instead of performing radiofluorination directly on the electron-rich benzene ring was to increase the radiolabeling yield. Although iodonium and pinacol-borate were both introduced for aryl radiofluorination, the yield still varied greatly depending on the substituent. For example, [^18^F]MFBG without any electron-donating substituent could obtain 6–31% RCY depending on the structure of the precursor [33,34,35]. Among them, the highest RCY (31%) was obtained when using the precursor with a fully-protected guanidine and iodonium ylide precursor [35]. However, when an electron-donating hydroxyl group exists on the benzene-ring, such as in the case of [^18^F]MHPG and PHPG, using spirocyclic iodonium ylide precursors, the RCYs decreased to approximately 8% on average, also when using an iodonium precursor [36,37]. In contrast, using the newly developed pinacol-borate precursors, several electron-rich tracers with protected phenolic hydroxyl groups could be radiofluorinated with reasonable yield. Typical examples include 6F-dopamine (20–29% RCY) and 6F-DOPA (22% RCY) [38].

As proven by the competitive cellular uptake study with the cold reference compounds, an obvious conclusion of this study is that the interaction between a radiotracer and the NET is strongly and positively correlated to the physicochemical property of the *meta*-substituent in vitro (Table 1, Figure 3G). AF78(F), bearing the most electronegative halogen atom in the *meta*-position, showed the best NET affinity, which is comparable to the physiological neurotransmitter NE. As the electronegativity decreased among the halogens, the affinities of AF78(Cl) and (Br) with *meta*-chlorine and *meta*-bromine substituents also dropped. As the least electronegative halogen applied, AF78(I) with a *meta*-iodine, was 15-fold less potent than AF78(F). In accordance with the trend observed in the cellular assay, the H/B ratio of the tracers also decreased as the *meta* substituent changed from fluorine to iodine, which strongly indicated higher NET-mediated uptake with increased electronegativity, which is inverse to the size of the halogen. That is, if the remainder of the compound remains unchanged, the molecule containing a *meta*-substituent with higher electronegativity and a smaller atomic size shows higher NET affinity (Table 1, Figure 3G). Indeed, instead of solely depending on the NET affinities of these cold references, an argument can be raised that the competitive cellular uptake of the tested compounds against [^3^H]NE is a combination of multiple factors including NET-specific transportation, uptake into storage vesicles and passive efflux from the cells. First, the uptake of AF78(F) specifically mediated by NET has been proven experimentally, and the compounds examined in the current study are analogues of it [17]. Second, it has been proven that storage vesicles in the SK-N-SH cell line are rare [39], so no extra transport into these vesicles via VMAT2 needs to be included. Third, although the NET tracer transported into the cell is stored in the cytosol, the passive efflux ratio is extremely slow compared with the effective NET-mediated transport, cf. the efflux of LMI1195, a structure with benzylguanidine and 3-fluoropropyl moieties [40]. As a result, only the NET-mediated specific uptake contributes to the competitive uptake studies, and the results can be considered as a reflection of the radiotracers’ NET affinity.

However, although these compounds bear an aliphatic “tail” structure, it is reasonable that molecules targeting NET may adopt a different binding pose, i.e., different SARs. On the one hand, most NET inhibitors are tricyclic structures with very bulky lipophilic backbones that interact with the NET binding pockets. This was observed in the AF78 series of tracers, as *meta* halogen substituents showed better affinities, and AF78(OH) had the lowest affinity with the exception of the unsubstituted tracer AF78(H). On the other hand, this presumption seems to contradict the fact that several NET-targeting radiotracers, such as *meta*-hydroxyephedrine (HED) or 6-fluorodopamine (6F-DA), along with NE, all contain phenolic OH groups at *meta* and/or *para* positions (Figure 1), which seemed crucial for NET affinity. It is noteworthy that, although bearing such structural differences, FBBG showed global and regional distribution comparable to those of HED in both healthy human subjects and in patients with ischemic cardiomyopathy, according to a study published in 2021 [16]. A *meta*-hydroxyl group/phenol is normally not physiologically stable and quickly undergoes phase II metabolism in vivo, namely, sulfation and glucuronidation [41]. The addition of fluorine to a benzene ring is generally considered a metabolically stable bioisostere of phenol. Therefore, a fluorine would be the ideal substituent at this position, which has been shown in the structural isomers 4-fluoro-3-hydroxyphenethylguanidine (4F-MHPG) and 3-fluoro-4-hydroxyphenethylguanidine (3F-PHPG) (Figure 1). Nevertheless, such a pair of isomers does not apply to AF78(F) and AF78(OH) in the current study since the former was the most active and the latter was the least active compound tested (Table 1). Therefore, considering the contradiction mentioned above, SARs should not be simplified; instead, one should presume that when a molecule interacts with the binding site of NET, it may adopt a totally different binding pose and represent an opposite trend in affinity when comparing the “smaller” molecules, e.g., HED or 6F-DA, and the “bigger” and bulkier molecules, such as tracers with a “tail” or tricyclic antidepressants. For example, the MIBG analog 4-[^18^F]fluoropropoxy-3-iodobenzylguanidine (FPOIBG) reported by Vaidyanathan et al. used a similar strategy to introduce a “tail” moiety for radiofluorination, and the resulting tracer showed less favorable properties than MIBG and FBBG in both in vitro and in vivo assays [42]. When considering the loss of NET affinity of our previously reported tracer candidate, 1-(4-fluoro-3-(3-fluoropropoxy)-phenethylguanidine with a *meta*-fluoropropoxyl group derived from MHPG [17], one may attribute this loss to steric hindrance after the introduction of such a bulky group. However, size may not be the only explanation for such affinity changes when comparing AF78(OH) with AF78(Br) and AF78(I). The atomic volume of a hydroxyl group is smaller than the volumes of the aforementioned halogens, but the affinity of AF78(OH) was far lower than that of the latter two molecules.

It is common in drug discovery and development to quantitatively rationalize the interaction of molecules with the binding site of the target protein using computational modeling and docking studies [43]. Unfortunately, until now, there has been no available *X*-ray structure of a crystalized *h*NET. Hence, the modeling of NET and docking of NET-targeting molecules, mostly for the development and rationalization of NET inhibitors (namely, antidepressants), uses surrogate structures, which is exemplified by the model proposed by Pidathala et al. using *h*NET-like mutations from the dopamine transporter PDB6M0 [28]. We consequently used this crystal structure as a surrogate model for the exploration of the binding modes of the listed compounds via a molecular docking approach. In this structure, three binding pockets were introduced to predict the binding poses and affinities of the NET inhibitors relevant to this publication, which are displayed in Figure 3. In a model based on the leucine transporter LeuT from *Aquifex aeolicus*, Schlessinger et al. demonstrated that there is no interaction between the *meta*-hydroxy of NE and Y152 of the NET [44]. In addition, there is limited space between the *meta* position and Y152, which allows for only minor changes to the design of NET radiotracers. Both MIBG and FBBG have been proven in human studies [5]. They share a benzylguanidine core structure and are, therefore, not exposed to the same spatial stress as this AF78 series. The sweeping loss in affinity of the hydroxy substituent may be explained by an additive effect of repulsion into this hydrophobic hinge and the space taken by this functional group. As a result, considering both the electrostatic and exchange-repulsion, the strong electron-withdrawing fluorine in AF78(F) is most favorable. Such an effect has been quantitatively proven by Sinnokrot et al., who showed increased T-shaped π–π interactions with the electron-withdrawing effects of the substituents H, OH, CH_3_, F and CN in sequence [45].

Finally, encouraged by the results obtained from both the cell-based studies and in vivo assessment in rodents, namely, specific uptake in the heart and brown adipose tissue (Figure 2C), NET imaging in NHPs using AF78(F) was performed as the first step in translational studies to prepare for potential clinical evaluation. Distinct cardiac uptake comparable to rodents was observed in the NHPs. However, in contrast to the high heart-to-liver ratio noted in rodents, slightly higher liver uptake was also observed (Figure 2D) [18], which is in accordance with other high-affinity NET-targeting radiotracers, such as MIBG and HED. For further detailed kinetics of AF78(F) in NHPs, please refer to Reference 18. One possible explanation for this finding could be nonspecific uptake via organic cation transporters [46]. From a structural point of view, either the secondary amine of HED or the guanidine moieties of MIBG and AF78(F) form positively charged cations under physiological conditions, making them potential substrates of organic cation transporters. Clearly, species differences are responsible for such variations and are crucial components that should be considered in translational medicine since many factors, such as metabolism and protein expression, are inconsistent between species and deserve attention during the development of both radiotracers and therapeutic medications. Further evaluation in NHPs is ongoing to further understand the characteristics of the AF78 series of tracers, which will be helpful for the further development of NET-targeting tracers with better NET selectivity over organic cation transporters. Moreover, the toxicity of AF78(F) is currently under evaluation before further clinical studies.

A limitation of the current study is a lack of even broader structural variety with the small number of compounds that were synthesized and examined with a major focus on the *meta*-substituent of AF78 series of compounds. Therefore, the results, and in particular the computational studies, should only be generalized with caution. Further evaluation with a large variety of structures and including all reported NET tracers using transfected NET-overexpressing HEK cell lines should be performed. Indeed, the result of the competitive cell uptake study is the combination of a complex process including NET binding, transporting, and possible efflux from the cytosol and it provides information closer to reality than a simple binding assay.

## 5. Conclusions

A series of analogs of AF78 were synthesized and evaluated both in vitro and in vivo. Based on the results, the SARs were characterized and compared with the computational modeling established to date. For the interaction of a NET-targeting radiotracer with a “tail” structure, a T-shaped π–π stacking interaction between the benzene ring of the radiotracer and the tyrosine and phenylalanine residues surrounding the NET binding site seems crucial and is positively correlated with the electronegativity of the *meta*-substituent on the benzene ring. This study is an initial venture undertaken to rationalize the interaction between NET-targeting radiotracers and their binding sites, which will prove helpful for further optimization of tracer structures. The SARs presented here provide a rationale for the high affinity of AF78(F), as demonstrated by cardiac imaging in rat and NHP hearts, and further evaluation in animals for translational studies is in progress to better understand the characteristics of this tracer.

## Figures and Tables

**Table 1:** Chemical structures, competitive cellular uptake activities and physical properties of the reference compounds, cold references and corresponding radiolabeling precursors.

Name	Cold References	IC_50_ Values (µM) ^§^	Physical Properties of Halogen Substitution			Radiolabeling Precursors		
	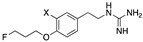		Electronegativity According to the Pauling Scale [19]	Atomic Radius (Å) [20]	Atomic Volume (Relative to H) [21]	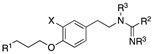		
	X =					R^1^ =	R^2^ =	R^3^ =
AF78(F)	F	0.42 ± 0.14 ^n.s.^	3.98	0.57	0.6	Tosyl	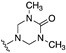	Boc
AF78(Cl)	Cl	0.94 ± 0.28 ^n.s.^	3.16	1.02	2.9	Brosyl	NHBoc	Boc or H
AF78(Br)	Br	3.32 ± 0.72 ***	2.96	1.20	4.4	Brosyl	NHBoc	Boc or H
AF78(I)	I	6.51 ± 3.32	2.66	1.39	6.9	Tosyl	NHBoc	Boc
AF78(H)	H	4.17 ± 0.92 ***	2.20	0.31	1.0	-	-	-
AF78(OH)	OH	22.67 ± 3.58	1.30 ^#^	1.10 ^†^	-	-	-	-
NE	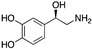	0.50 ± 0.16	-	-	-	-	-	-
DMI	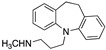	0.010 ± 0.0015 *	-	-	-	-	-	-
MIBG	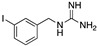	1.75 ± 0.47 **	-	-	-	-	-	-

^§^ IC_50_ values were measured in neuroblastoma cells at different concentrations vs. the uptake of [^3^H]NE. The results are presented as the mean ± SD for individual assays (n = 4) and compared with NE, where n.s. indicates *p* > 0.05; * *p* ≤ 0.05; ** *p* ≤ 0.01; *** *p* ≤ 0.0001. NE = norepinephrine, DMI = desipramine, MIBG = *meta*-iodobenzylguanidine. ^#^ Calculated from the difference in electronegativity between oxygen (3.5) and hydrogen (2.2). ^†^ Ionic radius of hydroxyl group [22].

## Data Availability

All experimental data have been included in the current manusciript.

## Supplementary Materials

The following supporting information can be downloaded at: https://www.mdpi.com/article/10.3390/pharmaceutics15020690/s1, Figure S1: Synthesis of cold derivatives AF78(Cl) & AF78(Br); Figure S2: Synthesis of cold derivative AF78(H); Figure S3: Synthesis of cold derivative AF78(I); Figure S4: Synthesis of cold derivative AF78(OH); Figure S5: Synthesis of labelling precursors for [^18^F]AF78(Cl) & [^18^F]AF78(Br); Figure S6: Synthesis of labelling precursors for [^18^F]AF78(I); Figure S7: Analytical HPLC chromatographies of [^18^F]AF78(Cl); Figure S8: Analytical HPLC chromatographies of [^18^F]AF78(Br); Figure S9: Analytical HPLC chromatographies of [^18^F]AF78(I); Figure S10: Dose response curves of [^3^H]NE uptake in SK-N-SH cells in the presence of increasing concentrations of nonradioactive AF78 derivatives. Table S1: Overview of the different precursor structures and the conditions applied for labeling AF78(Cl). Refs. [47,48,49] are mentioned in Supplementary Materials.

## Abbreviations

NET	norepinephrine transporter
NE	norepinephrine
SNS	sympathetic nervous system
PD	Parkinson’s disease
MIBG	*meta*-iodobenzylguanidine
FDA	US Food and Drug Administration
FBBG	1-(3-bromo-4-(3-[^18^F]fluoropropoxy)benzyl)-guanidine
PET	positron emission tomography
SARs	structure-activity relationships
DMI	desipramine
NHPs	non-human primates
RCY	radiochemical yield
