# Supplementary material for: Rationalizing the Binding Modes of PET Radiotracers Targeting the Norepinephrine Transporter
Source: Pharmaceutics. 2023 Feb 17;15(2):690. doi: 10.3390/pharmaceutics15020690 (PMC9963373; doi:10.3390/pharmaceutics15020690)
Supplement: Supplementary file 1 [file pharmaceutics-15-00690-s001.zip › pharmaceutics-2197181-supplementary.pdf]

Supplementary Information Text

Radiolabeling protocol

Radiolabeling of AF78(F) was performed according to a previously described protocol [17, 18] . Whereas the general radiolabeling protocol of new analogues, i.e. AF78(Cl, Br, I), was as follow: [^{18}F]Fluoride trapped on a Sep-Pak® Accell Plus QMA Plus Light cartridge (Waters GmbH, Germany, preconditioned by Ethanol 10 mL, 0.5 M K_2CO_3 5 mL, water 10 mL) was first washed with distilled water (2 mL) to remove [^{18}O]H_2O. [^{18}F]Fluoride was then eluted from the cartridge with a mixture of Et_4NHCO_3 or Bu_4NHCO_3 (0.075 M, 80 µL), water (120 µL) and acetonitrile (400 µL) into a 1 mL conical vial. The solution was dried azeotropically under flowing nitrogen at 120 °C. Acetonitrile (2 × 0.5 mL) was added to the vial for further drying. Labeling was carried out by adding the precursor (1.0–1.3 mg) to dry acetonitrile (300 µL) and heating at 90 °C for 10–15 min. Hydrochloric acid (6 M or 4 M HCl, 300 µL) was added to the reaction mixture if protected guanidine precursors were used, and the reaction continued at 110 °C for 15–20 min. The mixture was cooled and neutralized with aq. NaOH (6 M, 300 µL), which was further diluted with 1 mL of a mixed solution of water and acetonitrile (3:7) and subjected to semipreparative HPLC for purification. The HPLC constituents were as follows: column: COSMOSIL 5C18-ARII 6.0ID × 150 mm, 5 µm; mobile phase A: Millipore water with 0.1% TFA; and mobile phase B: acetonitrile with 0.1% TFA. Elution conditions: 0–15 min, 30–60% B, 15–17 min 60–95% B, 17–20 min 95% B, 22–30 min 30% B at a flow rate of 1.5 mL/min. Thin-layer chromatography (TLC)-autoradiography conditions: Normal-phase TLC was conducted on silica gel 60 in a developing system of dichloromethane:methanol:30% ammonia = 3:1:0.1; Rf = 0.5.

Competitive Cellular Uptake Studies

Human neuroblastoma cell line SK-N-SH from metastatic bone marrow stably expressing NET were cultivated according to the instructions from the supplier (Sigma-Aldrich Chemie GmbH, Munich, Germany). One day before the competitive assay, the cells were seeded in 24-well plates at a density of 1 × 10^5 cells/well and incubated in 0.5 mL of Eagle's minimum essential medium (EMEM) at 37 °C overnight. The medium was removed, and the cells were washed with 0.5 mL of Dulbecco's modified Eagle's medium (DMEM) with low glucose containing 0.1% bovine serum albumin (BSA). After washing, DMEM/0.1% BSA (300 µL) was added along with 50 µL of pargyline (1 mM/1.9569 mg/mL in DMSO) and 50 µL of pyrogallol (0.2 mM/0.25 mg/mL in DMSO). In the presence of pyrogallol (20 µM final concentration, catechol-O-methyl transferase inhibitor) [1] and pargyline (100 µM final concentration, monoamine oxidase inhibitor) [2], 50 µL of working solution containing [^{3}H]NE (12 kBq/mL prepared from purchased solution with molar activity 1024.9 GBq/mmol, NET678250UC, PerkinElmer LAS GmbH, Germany) in DMEM/0.1% BSA together with 50 µL of the test compounds (desipramine, MIBG, HED and cold references, each at different concentrations ranging from 10^{-10}–10^{-4} M) was added to each well, resulting in 500 µL total incubation volume. The addition of the inhibitors of both enzymes involved in the natural metabolism of NE is necessary to prevent the radiotracer from decomposition during the incubation time. The plates were incubated at 37°C for 60 min. The cells were then washed with ice-cold PBS (2 × 0.5 mL) followed by the addition of NaOH solution (0.1 N, 500 µL) and collection of the cell lysates. Cell lysates (140 µL) were mixed with 1.8 mL of scintillation cocktail (Hionic-Fluor, PerkinElmer, Rodgau, Germany) and radioactivity was measured in a liquid scintillation analyzer (Tri-Carb 2810 TR, PerkinElmer, Rodgau, Germany). All experimental data are presented as mean ± SD, with individual numbers measured in triplicate in experiment performed on 2–3 separate

days. IC_{50} values were determined using nonlinear regression log(inhibitor) vs. normalized response in GraphPad Prism 8.4.3.

PET Imaging and Biodistribution Study

PET imaging of [^{18}F]AF78(F) in rodents (n = 2) was obtained using a dedicated small animal PET system (microPET Focus 120, Siemens, Germany). The PET imaging protocol was designed to assess the systemic and myocardial radiotracer distribution of [^{18}F]AF78(F). Shortly before the single injection of [^{18}F]AF78(F) (10–20 MBq) via the tail vein, a 10 min dynamic PET scan was initiated with acquisition in list-mode format. After the PET scan, anatomical CT images were acquired using a small animal SPECT/CT system (Bioscan NanoSPECT/CT, VitalScan, Santa Barbara, CA, US). For the rat biodistribution studies, radiotracers (1–2 MBq) were administered via the tail vein. Ten minutes after radiotracer administration, the animals were euthanized. The organs of interest were harvested for tissue counting with a γ-counter (2480 Automatic Gamma Counter WIZARD^2, PerkinElmer, Rodgau, Germany). Following weight and decay correction of the tissue counts, the heart-to-blood ratios were calculated. NHPs (n = 2) were anesthetized during the imaging study and studied on a clinical PET scanner (PCA-2000A, Toshiba Medical Systems Corporation, Tochigi, Japan). After a 5 min transmission scan and an intravenous injection of the radiotracer [^{18}F]AF78(F) (approximately 20 MBq), a 60-min dynamic PET scan (10 s × 12, 30 s × 6, and 300 s × 11) was started. For the blocking study, desipramine (DMI; 1 mg/kg) was intravenously injected 10 min before tracer administration, after which the PET scan started immediately. The data were sorted into 3-dimensional sinograms, which were then reconstructed to produce dynamic images using a 2-dimensional ordered-subset expectation maximization algorithm. All images were corrected for decay, random coincidence and dead time; correction for attenuation was performed for NHPs but not rats. The obtained PET images were analyzed with the public domain tool AMIDE imaging software (A Medical Imaging Data Examiner, version 1.01).

Synthesis and Radiolabeling

Different cold reference compounds (nonradioactive fluorine-19 compounds) of the *meta*-substituted analogs of AF78 were synthesized (Table 1). The preparation of AF78(Cl) and AF78(Br) was performed in seven steps, applying a Sandmeyer reaction as the key step to introduce a halogen atom via a common anilinic intermediate. Transformation of the aniline into a diazonium salt allowed radical-nucleophilic aromatic substitution by a chlorine or bromine atom under copper (I) catalysis (Supplemental Fig. 1), while the guanidine moiety was coupled using a Mitsunobu reaction. During the Sandmeyer reaction, partial dehalogenation was observed. In order to broaden SARs, the by-product AF78(H) was also isolated (Supplemental Fig. 2) and examined in the competitive cellular uptake study (Table 1). AF78(I) was obtained in nine steps following a different synthetic approach using 3-iodo-4-methoxybenzaldehyde as starting material, on which a *meta*-iodine substituent is already present (Supplemental Fig. 3). AF78(OH) was prepared starting from 4-hydroxy-3-methoxybenzaldehyde with selective demethylation being the key step, as it did not affect the 3-fluoropropyl ether group (Supplemental Fig. 4).

To synthesize the precursors for radiolabeling, the Sandmeyer strategy was not applied because of the chemically demanding linker preparation. The precursors of the chloride tracer with or without *tert*-butyloxycarbonyl (Boc) protecting groups on the guanidine moiety were synthesized to evaluate the possible influence on the radiolabeling procedure (Supplemental Fig. 5). The *meta*-iodine precursor was prepared in a total of nine steps by sharing the common intermediates of the cold reference for the first four steps (Supplemental Fig. 6).

The general protocol for radiolabeling was performed in a one-pot, two-step reaction, with nucleophilic radiofluorination followed by deprotection of the Boc-protected guanidine moiety under acidic conditions. The protocol was modified from the original protocol (17, 18) by replacing potassium carbonate/Kryptofix 222 with tetraethylammonium bicarbonate. This modification not only decreased the basicity of the radiolabeling mixture, affecting the stability of the precursors during the radiolabeling procedure, but also saved costs due to discontinuation of the use of the expensive reagent Kryptofix 222, which is preferable for potential clinical applications. *N,N',N"-tri*-Boc protection instead of the fully protected triazole guanidine was used in the present investigation (Fig. 3A) because an intermediate that had not been fully deprotected was found for the radiolabeling of AF78(F), thus lowering the labeling yield [17]. First, the radiolabeling of AF78(Br, Cl) from tri-Boc protected guanidine precursors does not show a critical influence in the radiolabeling condition: namely either Et_4NHCO_3/Bu_4NHCO_3 for the elution of $[^{18}F]F^-$ from QMA or different concentration of acid (4M/6M HCl) for deprotection. The isolated radiochemical yields (RCY) are 9.5 ± 2.8% and 0.5 ± 0.1% for AF78(Br) and (Cl), respectively. Second, in order to investigate the influence of protection group on guanidine to the RCY, precursor of AF78(Cl) with unprotected guanidine was prepared and the influence of radiolabeling conditions is presented in Table S1. The RCY using the Boc-protected guanidine precursor was very low under various labeling conditions. Different base to elute fluorine-18 from QMA cartrdige as well as reaction times showed little impact. By increasing the amount of precursor by 50% while decreasing the base to 3 equivalents, the yield changed just slightly. This might be due to the instability of the precursor under the radiolabeling conditions. After removing the Boc protecting group from the guanidine moiety with trifluoroacetic acid, the RCY indeed increased without further optimization (Table S1). It is noteworthy, the concentration of HCl added for the deprotection was not investigated. A possibility of the low RCY from precursors with tri-Boc protected guanidine is due to the decomposition of the labeled molecules in the harsh deprotection condition, i.e. 4–6 M HCl and 90°C heating. Removing the deprotection step by using precursor with unprotected guanidine

resulted in higher overall yields. Thorough investigation of the radiolabeling condition by using molder deprotection conditions is currently still ongoing.

The general radiolabeling procedure proved to be straightforward and could be finished manually within approximately 120 min, while for an unprotected guanidine, the time required for labeling could be even decreased to 90 min (Table S1). The average RCYs were 16 ± 6% (decay-corrected based on the starting radioactivity, n = 3–5) without optimization of the radiolabeling conditions. The molar activities of all the target radiotracers were >390 GBq/mmol and their radiochemical purities were >95% as confirmed by analytical high-performance liquid chromatography (HPLC) (**Supplemental Figs. 7−9**). The molar activity of AF78(F) used for animal studies was > 6.3 GBq/μmol.

After investigation of the radiolabeling process using precursors with a *tri*-Boc protected or a unprotected guanidine moiety, a one-step radiolabeling procedure using unprotected guanidine with shorter labeling times can be applied for future translational studies. Instead of using a fully protected guanidine moiety with a triazole structure as previously reported to radiolabel [^{18}F]AF78(F), in this work, we investigated how the structures of the precursors influence the RCY by using either *N,N',N"-tri*-Boc-protected guanidine precursors or, in the case of AF78(Cl), an unprotected precursor. When radiolabeling the *meta*-chlorine precursor, the best result was achieved using an unprotected guanidine moiety (10.0 ± 1.0%) without further protocol optimization. However, when using the same radiolabeling conditions for the *N,N',N"-tri*-Boc protected precursor, the reaction resulted in a 5-fold lower yield (2.6 ± 1.2%) even when using 50% more of the precursor together with the longer time period required for deprotection. Considering the resonance structure of guanidine, an explanation for this result could be that this group may not show sufficient nucleophilicity for radiofluorination, which is in contrast to the initial notion to use a precursor with a fully protected guanidine. Nevertheless, when using *N,N',N"-tri*-Boc-protected guanidine precursors, only the leftover BocNH can be deprotonated by the base used during radiofluorination, leading to a lower yield. As proven in the current study, an evident conclusion would be to use unprotected, rather than protected, precursors to save the time required for labeling when the labeling yield is not affected (Supplemental Table 1). Further optimization of the radiolabeling procedure using a precursor with an unprotected guanidine can be performed, paying particular attention to modify the precursor-to-base ratio.

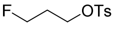 **3-Fluoropropyl 4-methylbenzenesulfonate (1)** 3-Fluoropropan-1-ol (1.5 g, 19.2 mmol, 1.0 eq.), 4-toluenesulfonyl chloride (4.0 g, 21.1 mmol, 1.1 eq.) and DMAP (0.23 g, 1.9 mmol, 0.1 eq.) were dissolved in dry DCM. Et_3N (4.0 mL, 28.8 mmol, 1.5 eq.) was added dropwise at 0 °C and the white suspension was stirred at r.t. for 14 h. The mixture was washed with water (20 mL) and extracted with CH_2Cl_2 (3 x 50 mL). The combined organic phases was washed with citric acid and saturated $NaHCO_3$ solution and dried over Na_2SO_4. The solvent was removed *in vacuo* to give the target compound as a colourless oil (3.6 g, 15.4 mmol, 81 %).
^{1}H NMR (400 MHz, $CDCl_3$): δ = 7.80 (d, *J* = 8.3 Hz, 1H), 7.36 (d, *J* = 8.3 Hz, 1H), 4.54 (t, *J* = 5.7 Hz, 1H), 4.42 (t, *J* = 5.7 Hz, 1H), 4.16 (t, *J* = 6.2 Hz, 1H), 2.45 (s, 2H), 2.11 – 2.04 (p, 1H), 2.00 (p, *J* = 11.8, 5.9 Hz, 1H) ppm. **ESI-MS**: m/z calcd. for $C_{10}H_{13}FO_3SH^+$ [M+H$^+$]: 233.1, found: 233.1.

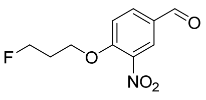

4-(3-Fluoropropoxy)-3-nitrobenzaldehyde (2) To a suspension of 4-hydroxy-3-nitrobenzaldehyde (1.42 g, 8.5 mmol, 1.0 eq.) and K_2CO_3 (1.76 g, 12.7 mmol, 1.5 eq.) in DMF (15 mL) was added a solution of 3-fluoropropyl 4-methylbenzenesulfonate (2.36 g, 10.2 mmol, 1.2 eq.) in DMF (3 mL) and the yellow solution was stirred at 70 °C for 16 h. After cooling to ambient temperature, the reaction mixture was diluted with water (50 mL) and extracted with EtOAc (3 x 30 mL). The combined organic layer was washed with saturated NaCl-solution (50 mL), dried over Na_2SO_4 and concentrated *in vacuo*. The product was isolated over column chromatography (PE:EtOAc = 3:1) as a yellow oil (1.74 g, 7.7 mmol, 90 %).
^{1}H NMR (400 MHz, $CDCl_3$): δ = 9.94 (s, 1H), 8.36 (d, *J* = 2.0 Hz, 1H), 8.08 (dd, *J* = 8.7, 2.1 Hz, 1H), 7.24 (d, *J* = 8.7 Hz, 1H), 4.75 (t, *J* = 5.6 Hz, 1H), 4.64 (t, *J* = 5.6 Hz, 1H), 4.36 (t, *J* = 6.0 Hz, 2H), 2.33 – 2.27 (p, 1H), 2.27 – 2.20 (p, 1H) ppm. **^{13}C NMR** (101 MHz, $CDCl_3$): δ = 188.9, 156.5, 134.8, 129.3, 127.6, 114.7, 80.01 (d, *J* = 165.1 Hz), 65.82 (d, *J* = 4.6 Hz), 30.10 (d, *J* = 20.1 Hz) ppm. **ESI-MS**: m/z calcd. for $C_{10}H_{10}FNO_4H^+$ [M+H$^+$]: 228.1, found: 242.1.

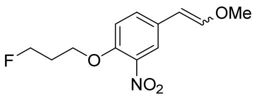

1-(3-Fluoropropoxy)-4-(2-methoxyvinyl)-2-nitrobenzene (3) To a solution of (methoxymethyl) triphenylphosphonium chloride (452 mg, 1.3 mmol, 1.5 eq.) in dry THF (10 mL) under argon atmosphere and 0 °C t-BuOK (197 mg, 1.8 mmol, 2.0 eq.) was added and the red reaction mixture was stirred for 2 min. Afterwards a solution of 4-(3-fluoropropoxy)-3-nitrobenzaldehyde (200 mg, 0.9 mmol, 1.0 eq.) in dry THF (5 mL) was added and the dark red reaction solution was stirred at r.t. for 18 h. Afterwards the solvent was removed *in vacuo* and the residue was portioned between water (20 mL) and EtOAc (20 mL) and the aqueous layer was extracted with EtOAc (2 x 20 mL). The combined organic layer was washed with saturated NaCl-solution (50 mL), dried over Na_2SO_4 and concentrated under reduced pressure. The product was isolated over column chromatography (PE:EtOAc = 6:1) as a yellow oil (188 mg, 0.7 mmol, 84 %).
^{1}H NMR (400 MHz, $CDCl_3$): δ = 8.09 (d, *J* = 2.2 Hz, 1H), 7.69 (d, *J* = 2.3 Hz, 1H), 7.65 (dd, *J* = 8.7, 2.2 Hz, 1H), 7.36 (dd, *J* = 8.7, 2.3 Hz, 1H), 7.02 – 6.97 (m, 3H), 6.17 (d, *J* = 6.9 Hz, 1H), 5.74 (d, *J* = 13.0 Hz, 1H), 5.16 (d, *J* = 6.9 Hz, 1H), 4.73 (dt, *J* = 8.4, 2.9 Hz, 2H), 4.61 (dt, *J* = 8.4, 2.9 Hz, 2H), 4.22 (dt, *J* = 6.0, 1.7 Hz, 4H), 3.80 (s, 3H), 3.69 (s, 3H), 2.27 – 2.20 (m, 2H), 2.20 – 2.14 (m, 2H) ppm. **^{13}C NMR** (101 MHz, $CDCl_3$): δ = 150.1, 149.8, 149.7, 148.7, 133.6, 130.6, 129.9, 129.4, 124.9, 121.9, 115.1, 114.3, 103.3, 102.9, 80.5 (d, *J* = 164.3 Hz), 80.4 (d, *J* = 164.4 Hz), 65.4 (d, *J* = 4.9 Hz), 65.2 (d, *J* = 5.0 Hz), 61.1, 56.9, 30.4 (d, *J* = 20.1 Hz). **ESI-MS**: m/z calcd. for $C_{12}H_{14}FNO_4H^+$ [M+H$^+$]: 256.1, found: 256.1.

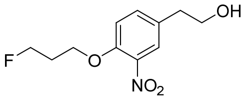

2-(4-(3-Fluoropropoxy)-3-nitrophenyl)ethan-1-ol (4) To a solution of 1-(3-fluoropropoxy)-4-(2-methoxyvinyl)-2-nitrobenzene (50 mg, 0.20 mmol, 1.0 eq.) in THF (2 mL) a solution of $Hg(OAc)_2$ (69 mg, 0.22 mmol, 1.1 eq.) in H_2O (4 mL) was added at 0 °C. The reaction solution was stirred for 10 min at 0 °C before a solution of $NaBH_4$ (30 mg, 0.78 mmol, 4.0 eq.) in saturated K_2CO_3 solution (2 mL) was added dropwise at 0°C. The reaction mixture was stirred for 30 min at 0 °C. Afterwards the mixture was filtered, and the filtrate diluted with water (15 mL) and the aqueous layer extracted with EtOAc (3 x 20 mL). The combined organic layer was washed with saturated NaCl-solution (40 mL), dried over Na_2SO_4 and concentrated *in vacuo*. The product was isolated over column chromatography (PE:EtOAc = 1:1) as a yellow oil (41 mg, 0.17 mmol, 86 %).
^{1}H NMR (400 MHz, $CDCl_3$): δ = 7.73 (d, *J* = 2.2 Hz, 1H), 7.41 (dd, *J* = 8.5, 2.2 Hz, 1H), 7.04 (d, *J* = 8.6 Hz, 1H), 4.74 (t, *J* = 5.6 Hz, 1H), 4.62 (t, *J* = 5.6 Hz, 1H), 4.22 (t, *J* = 6.0 Hz, 2H), 3.87 (t, *J* = 6.4 Hz, 2H), 2.86 (t, *J* = 6.4 Hz, 2H), 2.28 – 2.21 (p, 1H), 2.18 (p, *J* = 5.8 Hz, 1H) ppm. **^{13}C NMR** (101 MHz, $CDCl_3$): δ = 150.9, 139.9, 134.9, 131.7, 125.9, 114.8, 80.4 (d, *J* = 164.4 Hz), 65.3 (d, *J* = 4.9 Hz), 63.2, 37.8, 30.3 (d, *J* = 20.1 Hz) ppm. **ESI-MS**: m/z calcd. for $C_{11}H_{14}FNO_4H^+$ [M+H$^+$]: 244.1, found: 244.1.

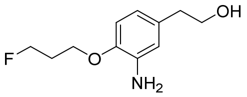

2-(3-Amino-4-(3-fluoropropoxy)phenyl)ethan-1-ol (5) A solution of 2-(3-nitro-4-(3-fluoropropoxy)phenyl)ethan-1-ol (300 mg, 1.23 mmol, 1.0 eq) in EtOH (6 mL) was heated to 50 °C before iron powder (276 mg, 4.93 mmol, 4.0 eq.) and a solution of NH_4Cl (264 mg, 4.93 mmol, 4.0 eq.) in water (2 mL) was added. The mixture was refluxed for 1h. After cooling down to room temperature the mixture was filtered over Celite®. The filtrate was diluted with water (15 mL) and extracted with CH_2Cl_2 (3 x 20 mL). The combined organic layer was dried over Na_2SO_4, filtered and concentrated. The title compound was isolated by column chromatography (CH_2Cl_2:MeOH:NH_3 = 99:1:0.1) as a pale oil (237 mg, 1.11 mmol, 90 %).
^{1}H NMR (400 MHz, $CDCl_3$): δ = 6.73 (t, *J* = 9.0 Hz, 1H), 6.59 (t, *J* = 2.4 Hz, 1H), 6.58 – 6.53 (m, 1H), 4.71 (t, *J* = 5.8 Hz, 1H), 4.59 (t, *J* = 5.8 Hz, 1H), 4.12 (t, *J* = 6.1 Hz, 2H), 3.79 (t, *J* = 6.5 Hz, 2H), 2.73 (t, *J* = 6.5 Hz, 2H), 2.26 – 2.19 (m, 1H), 2.19 – 2.12 (m, 1H) ppm. **^{13}C NMR** (101 MHz, $CDCl_3$): δ = 145.2, 136.5, 131.5, 118.9, 116.0, 112.0, 81.0 (d, *J* = 165.0 Hz), 64.3 (d, *J* = 5.2 Hz), 63.9, 38.7, 30.6 (d, *J* = 20.0 Hz) ppm. **ESI-MS**: m/z calcd. for $C_{11}H_{16}FNO_2H^+$ [M+H$^+$]: 214.1, found: 214.2.

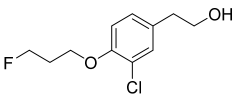

2-(3-Chloro-4-(3-fluoropropoxy)phenyl)ethan-1-ol (6(Cl)) To a solution of 2-(3-amino-4-(3-fluoropropoxy)phenyl)ethan-1-ol (50 mg, 0.23 mmol, 1.0 eq.) in H_2O (0.5 mL) slowly and under cooling conc. HCl (2 mL) was added. The solution was stirred under argon atmosphere at − 10 °C for 20 min before a cool solution of $NaNO_2$ (19 mg, 0.28 mmol, 1.2 eq.) in H_2O (1 mL) was slowly added to the bottom of the flask. The greenish solution was stirred for 20 min at room temperature before adding slowly to CuCl (70 mg, 0.70 mmol, 3.0 eq.) in conc. HCl (2 mL). The yellow solution was heated to 100 °C and stirred at this temperature for 1h before pouring over an ice-water solution (20 mL). The aqueous phase was extracted with CH_2Cl_2 (3 x 30 mL). The combined organic layer was washed with saturated $NaHCO_3$-solution (50 mL), dried over anhydrous Na_2SO_4 and concentrated *in vacuo*. Purification was performed by column

chromatography (PE:EtOAc = 3:1) to obtain a mixture of the title compound and the defunctionalized one (42 mg) as a pale oil.

^{1}H NMR (400 MHz, $CDCl_3$): δ = 7.24 (d, *J* = 2.1 Hz, 1H), 7.07 (dd, *J* = 8.3, 2.1 Hz, 1H), 6.88 (d, *J* = 7.0, 3.1 Hz, 1H), 4.75 (t, *J* = 5.7 Hz, 1H), 4.63 (t, *J* = 5.7 Hz, 1H), 4.14 (t, *J* = 6.0 Hz, 2H), 3.82 (t, *J* = 6.5 Hz, 2H), 2.79 (dd, *J* = 8.1, 4.9 Hz, 2H), 2.28 – 2.14 (m, 2H) ppm. **^{13}C NMR** (101 MHz, $CDCl_3$): δ = 153.1, 132.2, 130.8, 130.1, 128.6, 123.2, 114.8, 113.9, 80.7 (d, *J* = 164.4 Hz), 64.9 (d, *J* = 5.2 Hz), 63.6, 38.1, 30.5 (d, *J* = 20.1 Hz) ppm. **ESI-MS**: m/z calcd. for $C_{11}H_{14}ClFO_2Na^+$ [M+Na$^+$]: 255.1, found: 255.1.

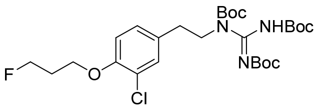

1-(4-(3-Fluoropropoxy)-3-chlorophenethyl)-*N,N',N"*-tri-Boc-guanidine (7(Cl)) A solution of 2-(3-chloro-4-(3-fluoropropoxy)phenyl)ethan-1-ol (125 mg, 0.54 mmol, 1.0 eq.), PPh_3 (211 mg, 0.81 mmol, 1.5 eq.) and *N,N',N"*-tri-Boc-guanidine (251 mg, 0.70 mmol, 1.3 eq.) in dry THF (7 mL) was cooled to 0 °C before a premixed solution of DEAD (126 µL, 0.81 mmol, 1.5 eq.) in THF (0.5 mL) was added. The solution was warmed to r.t. and stirred for 20 h. After removal of the solvent *in vacuo* the residue was dissolved in water (30 mL) and extracted with EtOAc (3 x 30 mL). The combined organic layer was washed with saturated NaCl-solution and dried over anhydrous Na_2SO_4. The product was isolated by column chromatography (PE:EtOAc = 30:1) as a colorless oil (104 mg, 0.18 mmol, 34 %).

^{1}H NMR (400 MHz, $CDCl_3$): δ = 10.57 (br s, 1H), 7.25 (s, 1H), 7.06 (dd, *J* = 8.3, 2.0 Hz, 1H), 6.84 (d, *J* = 8.4 Hz, 1H), 4.74 (t, *J* = 5.7 Hz, 1H), 4.62 (t, *J* = 5.7 Hz, 1H), 4.12 (t, *J* = 6.0 Hz, 3H), 3.99 – 3.94 (t, 2H), 2.92 – 2.85 (t, 2H), 2.26 – 2.19 (p, 1H), 2.19 – 2.13 (p, 1H), 1.51 (s, 18H), 1.46 (s, 9H) ppm. **^{13}C NMR** (101 MHz, $CDCl_3$): δ = 153.5, 152.9, 152.1, 132.7, 131.0, 128.4, 123.0, 113.7, 83.6, 80.7 (d, *J* = 164.3 Hz), 64.9 (d, *J* = 5.2 Hz), 49.1, 34.2, 30.5 (d, *J* = 20.1 Hz), 28.2, 28.1 ppm. **ESI-MS**: m/z calcd. for $C_{27}H_{41}ClFN_3O_7H^+$ [M+H$^+$]: 574.3, found: 574.3.

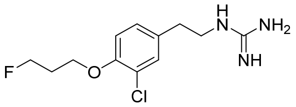

1-(3-Chloro-4-(3-fluoropropoxy)phenethyl)guanidine (AF78(Cl)) 1-(4-(3-Fluoropropoxy)-3-chlorophenethyl)-*N,N',N"*-tri-Boc-guanidine (104 mg, 0.18 mmol) was dissolved in dry CH_2Cl_2 (0.75 mL). At 0 °C TFA (0.25 mL) was added and the solution was stirred at r.t. for 18 h. Afterwards, the solvent was evaporated and the target compound was obtained as the corresponding TFA salt (40 mg, 0.10 mmol, 57 %).

^{1}H NMR (400 MHz, $CDCl_3$): δ = 9.75 (br s, 1H), 7.18 (s, 1H), 7.01 (d, *J* = 7.4 Hz, 1H), 6.87 (d, *J* = 8.3 Hz, 1H), 6.37 (br s, 2H), 4.74 (t, *J* = 5.7 Hz, 1H), 4.62 (t, *J* = 5.7 Hz, 1H), 4.12 (t, *J* = 6.0 Hz, 2H), 3.33 (s, 2H), 2.79 (t, *J* = 6.2 Hz, 2H), 2.19 (dp, *J* = 22.1, 5.8 Hz, 2H) ppm. **^{13}C NMR** (101 MHz, $CDCl_3$): δ = 157.1, 153.6, 130.5, 130.4, 128.1, 123.4, 114.0, 80.8 (d, *J* = 164.0 Hz), 64.9 (d, *J* = 5.1 Hz), 43.1, 33.8, 30.4 (d, *J* = 19.9 Hz) ppm. **ESI-MS**: m/z calcd. for $C_{12}H_{17}ClFN_3OH^+$ [M+H$^+$]: 274.1, found: 274.1.

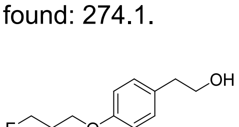

2-(3-Bromo-4-(3-fluoropropoxy)phenyl)ethan-1-ol (6(Br)) To a solution of 2-(3-amino-4-(3-fluoropropoxy)phenyl)ethan-1-ol (100 mg, 0.47 mmol, 1.0 eq.) in H_2O (1 mL) slowly and under cooling conc. HBr (3 mL) was added. The solution was stirred under argon atmosphere at − 10 °C for 20 min before a cool solution of $NaNO_2$ (39 mg, 0.56 mmol, 1.2 eq.) in H_2O (1 mL) was slowly added to the bottom of the flask. The greenish solution was added slowly

to CuBr (202 mg, 1.41 mmol, 3.0 eq.) in conc. HBr (3 mL). The violet solution was heated to 100 °C and stirred at this temperature for 1h before pouring over an ice-water solution (30 mL). The aqueous phase was extracted with CH_2Cl_2 (3 x 30 mL). The combined organic layer was washed with saturated $NaHCO_3$-solution (50 mL), dried over anhydrous Na_2SO_4 and concentrated *in vacuo*. Purification was performed by column chromatography (PE:EtOAc = 3:1) to obtain the title compound (90 mg, 0.33 mmol, 69 %) as a colourless oil.
^{1}H NMR (400 MHz, $CDCl_3$): δ = 7.42 (d, *J* = 1.9 Hz, 1H), 7.11 (dd, *J* = 8.3, 2.1 Hz, 1H), 6.86 (dd, *J* = 8.3, 3.6 Hz, 1H), 4.76 (t, *J* = 5.8 Hz, 1H), 4.64 (t, *J* = 5.8 Hz, 1H), 4.13 (t, *J* = 6.0 Hz, 2H), 3.82 (t, *J* = 6.5 Hz, 2H), 2.78 (t, *J* = 6.5 Hz, 2H), 2.24 (p, *J* = 5.9 Hz, 1H), 2.17 (p, *J* = 5.9 Hz, 1H) ppm. **^{13}C NMR** (101 MHz, $CDCl_3$): δ = 153.9, 133.9, 132.7, 129.1, 113.6, 112.5, 80.8 (d, *J* = 164.4 Hz), 64.9 (d, *J* = 5.2 Hz), 63.7, 38.0, 30.5 (d, *J* = 20.0 Hz) ppm. **ESI-MS**: m/z calcd. for $C_{11}H_{14}BrFO_2Na^+$ [M+Na$^+$]: 299.0, found: 298.9.

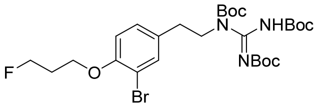

1-(4-(3-Fluoropropoxy)-3-bromophenethyl)-*N,N',N"*-tri-Boc-guanidine (7(Br)) A solution of 2-(3-bromo-4-(3-fluoropropoxy)phenyl)ethan-1-ol (85 mg, 0.31 mmol, 1.0 eq.), PPh_3 (121 mg, 0.46 mmol, 1.5 eq.) and *N,N',N"*-tri-Boc-guanidine (143 mg, 0.40 mmol, 1.3 eq.) in dry THF (5 mL) was cooled to 0 °C before a premixed solution of DEAD (72 µL, 0.46 mmol, 1.5 eq.) in THF (0.5 mL) was added. The solution was warmed to r.t. and stirred for 20 h. After removal of the solvent *in vacuo* the residue was dissolved in water (15 mL) and extracted with EtOAc (3 x 15 mL). The combined organic layer was washed with saturated NaCl-solution and dried over anhydrous Na_2SO_4. The product was isolated by column chromatography (PE:EtOAc = 20:1→10:1) as a colorless oil (59 mg, 0.10 mmol, 31 %).
^{1}H NMR (400 MHz, $CDCl_3$): δ = 7.43 (d, *J* = 1.9 Hz, 1H), 7.11 (dd, *J* = 8.3, 1.8 Hz, 1H), 6.81 (dd, *J* = 8.4, 3.6 Hz, 1H), 4.75 (t, *J* = 5.7 Hz, 1H), 4.63 (t, *J* = 5.7 Hz, 1H), 4.14 – 4.09 (m, 3H), 3.98 – 3.93 (m, 2H), 2.91 – 2.86 (m, 2H), 2.26 – 2.19 (m, 1H), 2.19 – 2.13 (m, 1H), 1.51 (s, 18H), 1.46 (s, 9H) ppm. **^{13}C NMR** (101 MHz, $CDCl_3$): δ = 153.6, 153.4, 133.8, 133.1, 129.0, 113.3, 112.1, 83.4, 80.8 (d, *J* = 164.4 Hz), 64.91 (d, *J* = 5.5 Hz), 60.4, 49.0, 33.9, 30.51 (d, *J* = 19.9 Hz), 28.1, 28.0, 27.9 ppm. **ESI-MS**: m/z calcd. for $C_{27}H_{41}BrFN_3O_7H^+$ [M+H$^+$]: 618.2, found: 618.2.

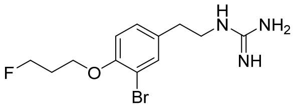

1-(3-Bromo-4-(3-fluoropropoxy)phenethyl)guanidine (AF78(Br)) 1-(4-(3-Fluoropropoxy)-3-bromophenethyl)-*N,N',N"*-tri-Boc-guanidine (46 mg, 0.07 mmol) was dissolved in dry CH_2Cl_2 (0.75 mL). At 0 °C TFA (0.25 mL) was added and the solution was stirred at r.t. for 18 h. Afterwards, the solvent was evaporated and the target compound was obtained as the corresponding TFA salt (29 mg, 0.07 mmol, 91 %).
^{1}H NMR (400 MHz, $CDCl_3$): δ = 7.46 (s, 1H), 7.34 (s, 1H), 7.05 (d, *J* = 7.5 Hz, 1H), 6.81 (d, *J* = 8.1 Hz, 2H), 5.08 (s, 1H), 4.73 (t, *J* = 5.6 Hz, 1H), 4.62 (t, *J* = 5.6 Hz, 1H), 4.09 (t, *J* = 5.9 Hz, 2H), 3.30 (s, 2H), 2.76 (s, 2H), 2.26 – 2.09 (m, 2H) ppm. **^{13}C NMR** (101 MHz, $CDCl_3$): δ = 154.3, 133.4, 131.3, 128.9, 113.7, 112.5, 80.8 (d, *J* = 164.1 Hz), 64.9 (d, *J* = 5.0 Hz), 33.7, 30.4 (d, *J* = 20.1 Hz) ppm. **ESI-MS**: m/z calcd. for $C_{12}H_{17}BrFN_3OH^+$ [M+H$^+$]: 318.1, found: 318.0.

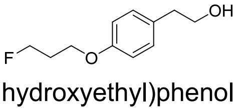

2-(4-(3-Fluoropropoxy)phenyl)ethan-1-ol (6(H)) To a suspension of 4-(2-hydroxyethyl)phenol (300 mg, 2.17 mmol, 1.0 eq.) and K_2CO_3 (451 mg, 3.26 mmol, 1.5 eq.) in DMF (5 mL) was added a solution of 3-fluoropropyl 4-methylbenzenesulfonate (606 mg, 2.61 mmol, 1.2

eq.) in DMF (1 mL) and the yellow solution was stirred at 70 °C for 16 h. After cooling to ambient temperature, the reaction mixture was diluted with water (30 mL) and extracted with EtOAc (3 x 30 mL). The combined organic layer was washed with saturated NaCl-solution (50 mL), dried over Na_2SO_4 and concentrated *in vacuo*. The product was isolated by column chromatography (PE:EtOAc = 3:1) as a white solid (309 mg, 1.56 mmol, 72 %).

^{1}H NMR (400 MHz, $CDCl_3$) δ 7.14 (d, *J* = 8.5 Hz, 2H), 6.86 (d, *J* = 8.4 Hz, 2H), 4.64 (dt, *J* = 47.1, 5.8 Hz, 2H), 4.08 (t, *J* = 6.1 Hz, 2H), 3.82 (t, *J* = 6.5 Hz, 2H), 2.81 (t, *J* = 6.5 Hz, 2H), 2.16 (dp, *J* = 25.8, 5.9 Hz, 2H) ppm. **^{13}C NMR** (101 MHz, $CDCl_3$): δ = 157.6, 130.8, 130.1, 114.8, 80.9 (d, *J* = 164.4 Hz), 64.0, 63.7 (d, *J* = 5.3 Hz), 38.4, 30.6 (d, *J* = 20.0 Hz). **ESI-MS**: m/z calcd. for $C_{11}H_{15}FO_2Na^+$ [M+Na$^+$]: 221.1, found: 221.1.

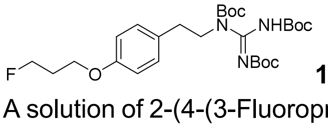

1-(4-(3-Fluoropropoxy)phenethyl)-*N,N',N''*-tri-Boc-guanidine (7(H)) A solution of 2-(4-(3-Fluoropropoxy)phenyl)ethan-1-ol (100 mg, 0.50 mmol, 1.0 eq.), PPh_3 (198 mg, 0.76 mmol, 1.5 eq.) and *N,N',N''*-tri-Boc-guanidine (235 mg, 0.66 mmol, 1.3 eq.) in dry THF (5 mL) was cooled to 0 °C before a premixed solution of DEAD (119 µL, 0.76 mmol, 1.5 eq.) in THF (1 mL) was added. The solution was warmed to r.t. and stirred for 20 h. After removal of the solvent *in vacuo* the residue was dissolved in water (15 mL) and extracted with EtOAc (3 x 15 mL). The combined organic layer was washed with saturated NaCl-solution and dried over anhydrous Na_2SO_4. The product was isolated by column chromatography (PE:EtOAc = 10:1) as a colorless oil (146 mg, 0.27 mmol, 54 %).

^{1}H NMR (400 MHz, $CDCl_3$): δ = 7.14 (d, *J* = 8.5 Hz, 2H), 6.81 (d, *J* = 8.5 Hz, 2H), 4.63 (dt, *J* = 47.1, 5.8 Hz, 2H), 4.06 (t, *J* = 6.1 Hz, 2H), 3.97 (dd, *J* = 8.5, 7.0 Hz, 2H), 2.93 – 2.87 (m, 2H), 2.15 (dp, *J* = 25.8, 6.0 Hz, 2H), 1.51 (s, 18H), 1.46 (s, 9H) ppm. **^{13}C NMR** (101 MHz, $CDCl_3$): δ = 157.5, 153.7, 152.0, 131.4, 130.2, 114.6, 83.4, 80.9 (d, *J* = 164.4 Hz), 63.7 (d, *J* = 5.3 Hz), 49.3, 34.3, 30.6 (d, *J* = 20.0 Hz), 28.3, 28.1 ppm. **ESI-MS**: m/z calcd. for $C_{27}H_{42}FN_3O_7H^+$ [M+H$^+$]: 540.3, found: 540.3.

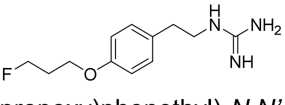

1-(4-(3-Fluoropropoxy)phenethyl)guanidine (AF78(H)) 1-(4-(3-Fluoropropoxy)phenethyl)-*N,N',N''*-tri-Boc-guanidine (50 mg, 0.09 mmol) was dissolved in dry CH_2Cl_2 (0.75 mL). At 0 °C TFA (0.25 mL) was added and the solution was stirred at r.t. for 18 h. Afterwards, the solvent was evaporated and the target compound was obtained as the corresponding TFA salt (32 mg, 0.09 mmol, 97 %).

^{1}H NMR (400 MHz, MeOD): δ = 7.19 (d, *J* = 8.6 Hz, 2H), 6.91 (d, *J* = 8.6 Hz, 2H), 4.63 (dt, *J* = 47.3, 5.9 Hz, 2H), 4.09 (t, *J* = 6.2 Hz, 2H), 3.43 (t, *J* = 7.1 Hz, 2H), 2.84 (t, *J* = 7.1 Hz, 2H), 2.15 (dp, *J* = 25.3, 6.0 Hz, 2H) ppm. **^{13}C NMR** (101 MHz, MeOD): δ = 159.3, 158.7, 131.4, 130.9, 115.8, 81.7 (d, *J* = 163.5 Hz), 64.8 (d, *J* = 5.5 Hz), 43.9, 35.1, 31.6 (d, *J* = 20.0 Hz) ppm. **ESI-MS**: m/z calcd. for $C_{12}H_{18}FN_3OH^+$ [M+H$^+$]: 240.1, found: 240.0.

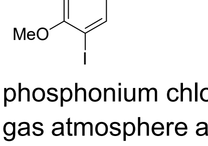

2-Iodo-1-methoxy-4-(2-methoxyvinyl)benzene (8) (Methoxymethyl)-triphenylphosphonium chloride (3.94 g, 11.50 mmol, 1.5 eq.) was dissolved in dry THF (60 mL) under inert gas atmosphere and KOtBu (1.47 g, 13.13 mmol, 1.7 eq.) was added at 0 °C. The reaction mixture was stirred for 2 min at 0 °C before 3-iodo-4-methoxybenzaldehyde (2.01 g, 7.66 mmol, 1.0 eq.) was added. The solution was stirred at room temperature for 18 h and evaporated to dryness. The

residue was partitioned between in EtOAc (50 mL) and water (50 mL) followed by layer separation. The aqueous layer was extracted with EtOAc (2 × 50 mL) and the combined organic layer was washed with saturated NaCl solution (100 mL) and dried over Na_2SO_4. The solvent was removed under reduced pressure and the crude material was purified by column chromatography (PE:EtOAc = 12:1) affording the desired product as a pale yellow oil (1.79 g, 6.17 mmol, 81% yield).
^{1}H NMR ($CDCl_3$, 400 MHz): δ = 8.02 (1H, d, *J* = 2.13 Hz), 7.66 (1H, d, *J* = 2.18 Hz), 7.50 (1H, dd, *J* = 2.13 Hz, 8.54 Hz), 7.15 (1H, dd, *J* = 2.25 Hz, 8.46 Hz), 6.92 (1H, d, *J* = 12.92 Hz), 6.78 - 6.71 (2H, m), 6.07 (1H, d, *J* = 6.94 Hz), 5.70 (1H, d, *J* = 13.00 Hz), 5.09 (1H, d, *J* = 6.98 Hz), 3.86/3.85 (6H, s), 3.77 (3H, s), 3.66 (3H, s) ppm. **^{13}C NMR** ($CDCl_3$, 101 MHz): δ = 156.4, 156.1, 148.5, 147.3, 139.1, 136.1, 131.3, 131.0, 129.4, 126.3, 111.2/110.7, 103.9, 103.4, 86.5, 86.0, 60.8, 56.7/56.6/56.5 ppm. **ESI-MS**: m/z calcd. for $C_{10}H_{11}IO_2H^+$ [M+H$^+$]: 291.0, found: 290.8.

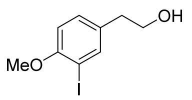

2-(3-Iodo-4-methoxyphenyl)ethan-1-ol (9) 2-Iodo-1-methoxy-4-(2-methoxy-vinyl)benzene (1.79 g, 6.17 mmol, 1.0 eq.) was suspended in THF (46 mL) and water (74 mL) before $Hg(OAc)_2$ (2.18 g, 6.84 mmol, 1.1 eq.) was added at 0 °C. The reaction mixture was stirred at 0 °C for 30 min followed by the addition of a 50% aqueous K_2CO_3 solution (30 mL) and $NaBH_4$ (942 mg, 24.90 mmol, 4.0 eq.). After the reaction mixture was stirred at 0 °C for 2.5 h, the precipitate was filtered off and the filtrate was extracted with EtOAc (3 × 80 mL). The combined organic layer was dried over Na_2SO_4 and the solvent was removed under reduced pressure. The crude material was purified by column chromatography (PE:EtOAc = 1:1) to obtain the alcohol as a yellow oil (1.36 g, 79% yield).
^{1}H NMR ($CDCl_3$, 400 MHz): δ = 7.65 (1H, d, *J* = 2.13 Hz), 7.16 (1H, dd, *J* = 2.14 Hz, 8.35 Hz), 6.76 (1H, d, *J* = 8.35 Hz), 3.85 (3H, s), 3.81 (2H, t, *J* = 6.55 Hz), 2.76 (2H, t, *J* = 6.53 Hz), 1.69 (1H, s) ppm. **^{13}C NMR** ($CDCl_3$, 101 MHz): δ = 157.0, 139.9, 132.9, 130.2, 111.1, 86.2, 63.7, 56.5, 37.8 ppm. **ESI-MS**: m/z calcd. for $C_9H_{11}O_2IH^+$ [M+H$^+$]: 278.9, found: 278.8.

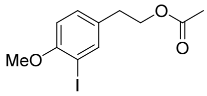

3-Iodo-4-methoxyphenethyl acetate (10) 2-(3-Iodo-4-methoxyphenyl)ethan-1-ol (1.04 g, 3.74 mmol, 1.0 eq.) was dissolved in acetone (20 mL) and acetyl chloride (800 µL, 11.21 mmol, 3.0 eq.) was added at 0 °C. The reaction mixture was stirred at room temperature for 18 h before the solvent was removed under reduced pressure. The residue was dissolved in EtOAc (20 mL) and washed with saturated aqueous $NaHCO_3$ solution (20 mL) and saturated NaCl-solution (20 mL). The organic layer was dried over Na_2SO_4 and the solvent was removed under reduced pressure. The crude material was purified by column chromatography (PE:EtOAc = 3:1) to obtain the acetate as a yellow oil (1.07 g, 3.34 mmol, 90% yield).
^{1}H NMR ($CDCl_3$, 400 MHz): δ = 7.63 (1H, d, *J* = 2.15 Hz), 7.15 (1H, dd, *J* = 2.16 Hz, 8.39 Hz), 6.75 (1H, d, *J* = 8.38 Hz), 4.22 (2H, t, *J* = 7.01 Hz), 3.85 (3H, s), 2.83 (2H, t, *J* = 7.01 Hz), 2.03 (3H, s) ppm. **^{13}C NMR** ($CDCl_3$, 101 MHz): δ = 171.1, 157.1, 139.9, 132.2, 130.0, 111.0, 86.1, 64.9, 56.5, 33.8, 21.1 ppm. **ESI-MS**: m/z calcd. for $C_{11}H_{13}O_3INa^+$ [M+Na$^+$]: 343.0, found: 342.9.

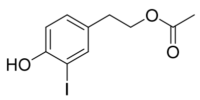

4-Hydroxy-3-iodophenethyl acetate (11) 3-Iodo-4-methoxyphenethyl acetate (588 mg, 1.87 mmol, 1.0 eq.) was dissolved in dry CH_2Cl_2 (12 mL) under inert gas atmosphere and a solution of BBr_3 (445 µL, 4.69 mmol, 2.5 eq.) in dry CH_2Cl_2 (12 mL) was added at −20 °C. The reaction mixture was stirred for 2 h and partitioned between EtOAc (60 mL) and a 50% aqueous

$NaHCO_3$ solution (60 mL). The layers were separated and the aqueous layer was extracted with EtOAc (2 × 60 mL). The combined organic layer was washed with saturated NaCl-solution (180 mL) and dried over Na_2SO_4. Evaporation of the solvent under reduced pressure and purification by column chromatography (PE:EtOAc = 3:1) afforded the phenol as a yellow oil (602 mg, 1.97 mmol, 92 % yield).

^{1}H NMR ($CDCl_3$, 400 MHz): δ = 7.51 (1H, d, *J* = 2.08 Hz), 7.08 (1H, dd, *J* = 2.05 Hz, 8.28 Hz), 6.91 (1H, d, *J* = 8.29 Hz), 5.47 (1H, bs), 4.22 (2H, t, *J* = 7.02 Hz), 2.83 (2H, t, *J* = 6.99 Hz), 2.04 (3H, s) ppm. **^{13}C NMR** ($CDCl_3$, 101 MHz): δ = 171.2, 153.8, 138.5, 132.0, 130.8, 115.1, 85.6, 65.0, 33.8, 21.1 ppm. **ESI-MS**: m/z calcd. for $C_{10}H_{11}O_3INa^+$ [M+Na^+]: 329.0, found: 328.9.

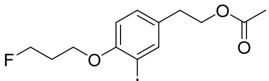

4-(3-Fluoropropoxy)-3-iodophenethyl acetate (12) 4-Hydroxy-3-iodophenethyl acetate (220 mg, 0.72 mmol, 1.0 eq.) and 3-fluoropropyl 4-methylbenzenesulfonate (158 mg, 0.68 mmol, 0.95 eq.) were dissolved in acetone (15 mL) and Cs_2CO_3 (468 mg, 1.44 mmol, 2.0 eq.) was added. The reaction mixture was stirred at 70 °C for 18 h before water (50 mL) was added. The mixture was extracted with EtOAc (3 × 50 mL) and the combined organic layer was washed with saturated NaCl-solution (150 mL) and dried over Na_2SO_4. The solvent was removed under reduced pressure and the crude material was purified by column chromatography (PE:EtOAc = 3:1) to give compound as a colourless oil (206 mg, 0.56 mmol, 79% yield).

^{1}H NMR ($CDCl_3$, 400 MHz): δ = 7.63 (1H, d, *J* = 2.15 Hz), 7.13 (1H, dd, *J* = 2.15 Hz, 8.30 Hz) 6.75 (1H, d, *J* = 8.33 Hz), 4.78 (1H, t, *J* = 5.79 Hz), 4.67 (1H, t, *J* = 5.78 Hz), 4.22 (2H, t, *J* = 7.00 Hz), 4.12 (2H, t, *J* = 5.79 Hz), 2.83 (2H, t, *J* = 6.97 Hz), 2.24 (1H, quint, *J* = 5.87 Hz), 2.18 (1H, quint, *J* = 5.87 Hz), 2.04 (3H, s) ppm. **^{13}C NMR** ($CDCl_3$, 101 MHz): δ = 171.1, 156.2, 139.8, 132.4, 130.0, 112.1, 86.7, 80.9 (d, *J* = 164.31 Hz), 64.9/64.8, 33.8, 30.5 (d, *J* = 20.19 Hz), 21.1 ppm. **ESI-MS**: m/z calcd. for $C_{13}H_{16}O_3IFH^+$ [M+H^+]: 367.0, found: 366.9.

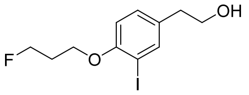

2-(4-(3-Fluoropropoxy)-3-iodophenyl)ethan-1-ol (6(I)) 4-(3-Fluoropropoxy)-3-iodophenethyl acetate (170 mg, 0.46 mmol, 1.0 eq.) was dissolved in MeOH (10 mL) and K_2CO_3 (642 mg, 4.64 mmol, 10.0 eq.) was added. The reaction mixture was stirred at room temperature for 3 h and was partitioned between water (50 mL) and EtOAc (50 mL) followed by layer separation. The aqueous layer was extracted with CH_2Cl_2 (2 × 50 mL) and the combined organic layer was dried over Na_2SO_4. The solvent was removed under reduced pressure to obtain the title compound as a yellow oil (165 mg, quant.) which was used directly in the next step.

^{1}H NMR ($CDCl_3$, 400 MHz): δ = 7.65 (1H, d, *J* = 2.13 Hz), 7.15 (1H, dd, *J* = 2.17 Hz, 8.31 Hz) 6.77 (1H, d, *J* = 8.31 Hz), 5.30 (1H, s), 4.78 (1H, t, *J* = 5.78 Hz), 4.67 (1H, t, *J* = 5.79 Hz), 4.12 (2H, t, *J* = 5.95 Hz), 3.81 (2H, t, *J* = 6.52 Hz), 2.77 (2H, t, *J* =6.49 Hz), 2.24 (1H, quint, *J* = 5.87 Hz), 2.18 (1H, quint, *J* = 5.86 Hz) ppm. **^{13}C NMR** ($CDCl_3$, 101 MHz): δ = 156.1, 139.9, 133.2, 130.2, 112.3, 87.0, 80.9 (d, *J* = 164.55 Hz), 64.9 (d, *J* = 5.22 Hz), 63.7, 37.9, 30.5 (d, *J* = 20.05 Hz) ppm. **ESI-MS**: m/z calcd. for $C_{11}H_{14}O_2IFH^+$ [M+Na^+]: 347.0, found: 346.9.

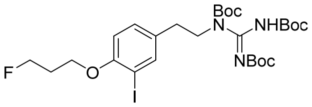

1-(4-(3-Fluoropropoxy)-3-iodophenethyl)-*N*,*N'*,*N''*-tri-*Boc*-guanidine (7(I)) 2-(4-(3-Fluoropropoxy)-3-iodophenyl)ethan-1-ol (165 mg, 0.51 mmol, 1.0 eq.), PPh_3 (201 mg, 0.77 mmol, 1.5 eq.) and *N*,*N'*,*N''*-tri-Boc-guanidine (263 mg, 0.73 mmol, 1.4 eq.)

were dissolved in dry THF (8 mL) under inert gas atmosphere. A solution of DIAD (151 µL, 0.77 mmol, 1.5 eq.) in dry THF (1.5 mL) was added at 0 °C and the reaction mixture was stirred at room temperature for 18 h. The solvent was removed under reduced pressure and the residue was partitioned between EtOAc (15 mL) and water (15 mL) followed by layer separation. The aqueous layer was extracted with EtOAc (2 × 15 mL) and the combined organic layer was washed with saturated NaCl-solution (45 mL) and dried over Na_2SO_4. The solvent was removed under reduced pressure and the crude product was purified by column chromatography (PE:EtOAc = 5:1) to give the title compound as a colourless oil (146 mg, 0.22 mmol, 53% yield).
^{1}H NMR ($CDCl_3$, 400 MHz): δ = 10.57 (1H, bs), 7.65 (1H, d, *J* = 2.16 Hz), 7.15 (1H, dd, *J* = 2.13 Hz, 8.30 Hz), 6.72 (1H, d, *J* = 8.35 Hz), 4.77 (1H, t, *J* = 5.80 Hz), 4.66 (1H, t, *J* = 5.80 Hz), 4.14 – 4.07 (3H, m), 4.00 – 3.91 (2H, m), 2.92 - 2.83 (2H, m), 2.23 (1H, quint, *J* = 5.92 Hz), 2.16 (1H, quint, *J* = 5.90 Hz), 1.51 (18 H, s), 1.46 (9H, s) ppm. **^{13}C NMR** ($CDCl_3$, 101 MHz): δ = 156.0, 153.6/152.0, 139.9, 133.8, 130.2, 112.1, 86.7, 83.6, 80.9 (d, *J* = 164.55 Hz), 64.9 (d, *J* = 5.22 Hz), 49.2, 33.8, 30.5 (d, *J* = 20.05 Hz), 29.9/28.3 ppm. **ESI-MS**: m/z calcd. for $C_{27}H_{41}N_3O_7IFH^+$ [M+H$^+$]: 666.2, found: 666.0.

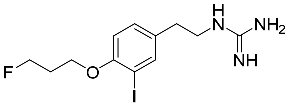

1-(4-(3-Fluoropropoxy)-3-iodophenethyl)guanidine (AF78(I)) 1-(4-(3-Fluoropropoxy)-3-iodophenethyl)-*N*,*N'*,*N''*-tri-Boc-guanidine (130 mg, 0.20 mmol, 1.0 eq.) was dissolved in dry CH_2Cl_2 (1 mL) and TFA (12 mL) was added. The reaction mixture was stirred at room temperature for 18 h before the solvent was removed under reduced pressure. However, LC-MS indicated the formation of a TFA amide. Therefore, the residue was dissolved in a mixture of water (3 mL) and MeOH (30 mL) prior to the addition of K_2CO_3 (165 mg, 1.19 mmol, 5.4 eq.). The reaction mixture was stirred at 80 °C for 2 h and was concentrated under reduced pressure. The aqueous residue was extracted with CH_2Cl_2 (4 × 5 mL) and the combined organic layer was dried over Na_2SO_4. The solvent was removed under reduced pressure and the crude product was purified by reversed phase flash column chromatography (RP-SiO2, Phase A: water with 0.1% formic acid, Phase B: methanol with 0.1% formic acid. Gradient B 5% for 5 min, 5 % to 40% in 35 min, 40% for 4 min, 40% to 100% in 15 min, 100% for 5 min, 100% to 90% in 1 min, 90% for 5 min) to give the target compound as a colourless oil (13 mg, 0.036 mmol, 18% yield).
^{1}H NMR ($CDCl_3$, 400 MHz): δ = 7.55 (1H, bs), 7.06 (1H, bs), 6.62 (1H, bs), 4.70 (1H, bs), 4.58 (1H, bs), 3.96 (2H, bs), 3.25 (2H, bs), 2.67 (2H, bs), 2.13 (1H, bs), 2.06 (1H, bs) ppm. **^{13}C-NMR** ($CDCl_3$, 101 MHz) δ = 157.5, 156.2, 139.5, 132.3, 130.1,112.3, 86.9, 81.0 (d, J = 163.90 Hz), 64.9 (d, J = 4.56 Hz), 43.1, 33.7, 30.4 (d, J = 19.96 Hz) ppm. **ESI-MS**: m/z calcd. for $C_{12}H_{17}N_3OIFH^+$ [M+H$^+$]: 366.0, found: 365.9.

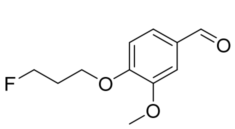

4-(3-Fluoropropoxy)-3-methoxybenzaldehyde (13) To a suspension of vanillin (500 mg, 3.29 mmol, 1.0 eq.) and K_2CO_3 (680 mg, 4.93 mmol, 1.5 eq.) in DMF (10 mL) was added a solution of 3-fluoropropyl 4-methylbenzenesulfonate (920 mg, 3.94 mmol, 1.2 eq.) in DMF (5 mL) and the yellow solution was stirred at 75 °C for 18 h. After cooling to ambient temperature, the reaction mixture was diluted with water (30 mL) and extracted with EtOAc (3 x 30 mL). The combined organic layer was washed with saturated NaCl-solution (50 mL), dried over Na_2SO_4 and concentrated *in vacuo*. The product obtained without further purification as a yellow oil (695 mg, quant.).

^{1}H NMR (400 MHz, $CDCl_3$): δ = 9.85 (s, 1H), 7.47 – 7.38 (m, 2H), 6.99 (d, *J* = 8.1 Hz, 1H), 4.67 (dt, *J* = 47.0, 5.7 Hz, 2H), 4.24 (t, *J* = 6.3 Hz, 2H), 3.91 (s, *J* = 4.5 Hz, 3H), 2.26 (dp, *J* = 26.4, 5.9 Hz, 2H) ppm. **^{13}C NMR** (101 MHz, $CDCl_3$): δ = 191.0, 153.9, 150.0, 130.4, 126.8, 111.8, 109.5, 80.60 (d, *J* = 164.9 Hz), 64.90 (d, *J* = 4.9 Hz), 56.1, 30.32 (d, *J* = 20.0 Hz) ppm. **ESI-MS**: m/z calcd. for $C_{11}H_{13}FO_3H^+$ [M+H$^+$]: 213.1, found: 213.2.

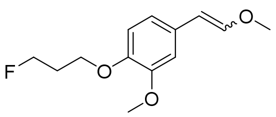

1-(3-Fluoropropoxy)-2-methoxy-4-(2-methoxyvinyl)benzene (14) To a solution of (methoxymethyl) triphenylphosphonium chloride (6.8 g, 19.80 mmol, 6.0 eq.) in dry THF (30 mL) under argon atmosphere and 0 °C t-BuOK (2.6 g, 23.10 mmol, 7.0 eq.) was added and the red reaction mixture was stirred for 2 min. Afterwards a solution of 4-(3-fluoropropoxy)-3-methoxybenzaldehyde (700 mg, 3.30 mmol, 1.0 eq.) in dry THF (5 mL) was added dropwise and the dark red reaction solution was stirred at r.t. for 5 min. Afterwards the solvent was removed *in vacuo* and the residue was portioned between water (40 mL) and EtOAc (40 mL) and the aqueous layer was extracted with EtOAc (2 x 40 mL). The combined organic layer was washed with saturated NaCl-solution (100 mL), dried over Na_2SO_4 and concentrated under reduced pressure. The product was isolated over column chromatography (PE:EtOAc = 3:1) as a yellow oil (640 mg, 2.66 mmol, 81 %).

^{1}H NMR (400 MHz, $CDCl_3$): δ = 7.24 (d, *J* = 1.9 Hz, 1H), 7.06 (dd, *J* = 8.3, 1.9 Hz, 1H), 6.94 (d, *J* = 12.9 Hz, 1H), 6.83 (dd, *J* = 8.2, 4.3 Hz, 2H), 6.79 – 6.73 (m, 2H), 6.07 (d, *J* = 7.0 Hz, 1H), 5.77 (d, *J* = 12.9 Hz, 1H), 5.17 (d, *J* = 7.0 Hz, 1H), 4.75 – 4.58 (m, 4H), 4.14 (dd, *J* = 11.8, 6.1 Hz, 4H), 3.86 (s, 6H), 3.77 (s, 3H), 3.67 (s, 3H), 2.27 – 2.13 (m, 4H) ppm. **^{13}C NMR** (101 MHz, $CDCl_3$): δ = 149.9, 149.3, 148.1, 146.8, 146.7, 146.5, 130.1, 129.8, 129.7, 127.2, 121.1, 117.8, 114.3, 113.6, 112.4, 109.1, 105.6, 105.0, 81.1 (d, *J* = 164.2 Hz), 81.0 (d, *J* = 164.3 Hz), 65.3 (d, *J* = 5.2 Hz), 65.1 (d, *J* = 5.2 Hz), 60.7, 56.6, 56.1, 56.0, 42.1, 30.7 (d, *J* = 1.3 Hz), 30.5 (d, *J* = 1.4 Hz) ppm. **ESI-MS**: m/z calcd. for $C_{13}H_{17}FO_3H^+$ [M+H$^+$]: 241.1, found: 241.2.

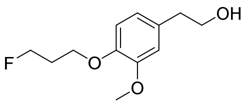

2-(4-(3-Fluoropropoxy)-3-methoxyphenyl)ethan-1-ol (6(OMe)) To a solution of 1-(3-fluoropropoxy)-2-methoxy-4-(2-methoxyvinyl)benzene (640 mg, 2.66 mmol, 1.0 eq.) in THF/H_2O (18/30 mL), $Hg(OAc)_2$ (934 mg, 2.93 mmol, 1.1 eq.) was added at 0 °C. The reaction solution was stirred for 10 min at 0 °C before a solution of $NaBH_4$ (400 mg, 10.66 mmol, 4.0 eq.) in saturated K_2CO_3 solution (15 mL) was added dropwise at 0°C. The reaction mixture was stirred for 30 min at 0 °C. Afterwards the mixture was filtered, and the filtrate diluted with water (30 mL) and the aqueous layer extracted with EtOAc (3 x 40 mL). The combined organic layer was washed with saturated NaCl-solution (100 mL), dried over Na_2SO_4 and concentrated *in vacuo*. The product was isolated over column chromatography (PE:EtOAc = 1:1) as a yellow oil (427 mg, 1.87 mmol, 70 %).

^{1}H NMR (400 MHz, $CDCl_3$): δ = 6.85 (d, *J* = 8.7 Hz, 1H), 6.75 (d, *J* = 6.8 Hz, 2H), 4.66 (dt, *J* = 47.1, 5.8 Hz, 2H), 4.13 (t, *J* = 6.3 Hz, 2H), 3.89 – 3.81 (m, 5H), 2.81 (t, *J* = 6.5 Hz, 2H), 2.20 (dp, *J* = 26.1, 6.0 Hz, 2H) ppm. **^{13}C NMR** (101 MHz, $CDCl_3$): δ = 149.79, 147.08, 131.79, 121.18, 114.00, 113.01, 81.02 (d, *J* = 164.3 Hz), 65.17 (d, *J* = 5.1 Hz), 63.84, 56.10, 38.90, 30.61 (d, *J* = 19.9 Hz) ppm. **ESI-MS**: m/z calcd. for $C_{12}H_{17}FO_3H^+$ [M+H$^+$]: 229.1, found: 229.1.

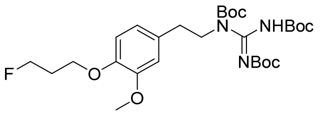

1-(4-(3-fluoropropoxy)-3-methoxyphenethyl)-*N,N',N''*-tri-Boc-guanidine (7(OMe)) A solution of 2-(4-(3-Fluoropropoxy)-3-methoxyphenyl)ethan-1-ol (200 mg, 0.88 mmol, 1.0 eq.), PPh_3 (346 mg, 1.31 mmol, 1.5 eq.) and *N,N',N''*-tri-Boc-guanidine (472 mg, 1.31 mmol, 1.3 eq.) in dry THF (10 mL) was cooled to 0 °C before a premixed solution of DEAD (345 µL, 1.75 mmol, 1.5 eq.) in THF (1 mL) was added. The solution was warmed to r.t. and stirred for 20 h. After removal of the solvent *in vacuo* the residue was dissolved in water (15 mL) and extracted with EtOAc (3 x 15 mL). The combined organic layer was washed with saturated NaCl-solution and dried over anhydrous Na_2SO_4. The product was isolated by column chromatography (PE:EtOAc = 6:1) as a colorless oil (223 mg, 0.39 mmol, 45 %).

^{1}H NMR (400 MHz, $CDCl_3$): δ = 6.83 – 6.77 (m, 2H), 6.73 (dd, *J* = 8.2, 1.7 Hz, 1H), 4.65 (dt, *J* = 47.1, 5.8 Hz, 2H), 4.12 (t, *J* = 6.3 Hz, 2H), 3.99 (dd, *J* = 8.7, 6.8 Hz, 2H), 3.84 (s, 3H), 2.95 – 2.87 (m, 2H), 2.19 (dp, *J* = 26.1, 6.0 Hz, 2H), 1.50 (s, 20H), 1.46 (s, 9H) ppm. **^{13}C NMR** (101 MHz, $CDCl_3$): δ = 153.6, 152.1, 149.7, 146.9, 132.5, 121.2, 113.8, 113.0, 83.5, 81.0 (d, *J* = 164.3 Hz), 65.2 (d, *J* = 5.2 Hz), 56.0, 49.3, 34.8, 30.6 (d, *J* = 19.9 Hz), 28.2, 28.1 ppm. **ESI-MS**: m/z calcd. for $C_{28}H_{44}FN_3O_8H^+$ [M+H$^+$]: 570.3, found: 570.3.

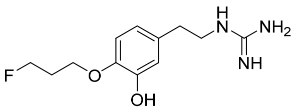

1-(4-(3-Fluoropropoxy)-3-hydroxyphenethyl)guanidine (AF78(OH)) The starting material (15 mg, 26 µmol) was dissolved in AcOH/HBr (2 + 0.2 mL) and heated to 110 °C for 3 h in a reaction vessel. After full deprotection, the solvent was removed under vacuum and the product isolated by preparative TLC (10 % MeOH in CH_2Cl_2) as the HBr salt (1.9 mg, 5.7 µmol, 22 %).

^{1}H NMR (400 MHz, MeOD): δ = 6.91 – 6.81 (m, 1H), 6.78 – 6.71 (m, 1H), 6.71 – 6.65 (m, 1H), 4.72 – 4.55 (m, 2H), 4.16 – 4.07 (m, 2H), 3.40 (t, *J* = 7.0 Hz, 2H), 2.80 – 2.71 (m, 2H), 2.23 – 2.09 (m, 2H) ppm. **ESI-MS**: m/z calcd. for $C_{12}H_{18}FN_3O_2H^+$ [M+H$^+$]: 256.1, found: 256.1.

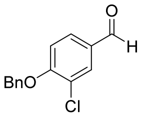

4-(Benzyloxy)-3-chlorobenzaldehyde (15(Cl)) To a suspension of 3-chloro-4-hydroxy benzaldehyde (5.00 g, 31.93 mmol, 1.0 eq.) and K_2CO_3 (8.83 g, 63.86 mmol, 2.0 eq.) in DMF (50 mL) at 0 °C BnBr (4.4 mL, 36.72 mmol, 1.2 eq.) was added dropwise under Argon. The reaction mixture was stirred at r.t. for 18 h. Afterwards, it was proportionate between water (50 mL) and EtOAc (100 mL). The organic layer was washed with water (2 x 80 mL) and with saturated NaCl-sol. (2 x 50 mL). The organic layer was dried over anhydrous Na_2SO_4, filtered and concentrated under reduced pressure to afford the title compound as a white solid (7.88 g, 31.93 mmol, quant.).

^{1}H NMR (400 MHz, $CDCl_3$): δ = 9.85 (s, 1H), 7.93 (d, *J* = 2.0 Hz, 1H), 7.73 (dd, *J* = 8.5, 2.0 Hz, 1H), 7.40 (ddt, *J* = 9.6, 7.2, 5.6 Hz, 5H), 7.08 (d, *J* = 8.5 Hz, 1H), 5.26 (s, 2H) ppm. **ESI-MS**: m/z calcd. for $C_{14}H_{11}ClO_2H^+$ [M+H$^+$]: 247.0, found: 247.0.

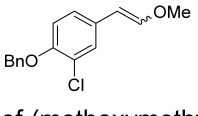

1-(Benzyloxy)-2-chloro-4-(2-methoxyvinyl)benzene (16(Cl)) To a suspension of (methoxymethyl) triphenylphosphonium chloride (16.43 g, 47.9 mmol, 1.5 eq.) in dry THF (130

mL) under argon atmosphere and 0 °C t-BuOK (7.17 g, 63.9 mmol, 2.0 eq.) was added and the red reaction mixture was stirred for 2 min. Afterwards a solution of 4-(benzyloxy)-3-chlorobenzaldehyde (7.88 g, 31.9 mmol, 1.0 eq.) in dry THF (30 mL) was added and the dark red reaction solution was stirred at r.t. for 18 h. Afterwards the solvent was removed *in vacuo* and the residue was portioned between water (70 mL) and EtOAc (70 mL) and the aqueous layer was extracted with EtOAc (2 x 70 mL). The combined organic layer was washed with saturated NaCl-solution (120 mL), dried over Na_2SO_4 and concentrated under reduced pressure. The product was isolated as a mixture over column chromatography (PE:EtOAc = 6:1) as a colourless oil (3.62 g, 13.18 mmol, 41 %).
^{1}H NMR (400 MHz, $CDCl_3$): δ = 7.69 (d, *J* = 2.1 Hz, 1H), 7.46 (d, *J* = 7.4 Hz, 4H), 7.42 – 7.36 (m, 5H), 7.33 (ddd, *J* = 8.5, 4.5, 2.6 Hz, 3H), 7.27 (d, *J* = 2.2 Hz, 1H), 7.02 (dd, *J* = 8.5, 2.2 Hz, 1H), 6.93 (d, *J* = 13.0 Hz, 1H), 6.87 (dd, *J* = 8.5, 4.7 Hz, 2H), 6.09 (d, *J* = 7.0 Hz, 1H), 5.71 (d, *J* = 13.0 Hz, 1H), 5.14 (d, *J* = 5.6 Hz, 4H), 5.11 (d, *J* = 7.0 Hz, 1H), 3.77 (s, 3H), 3.67 (s, 3H) ppm. **^{13}C NMR** (101 MHz, $CDCl_3$): δ = 152.3, 152.1, 148.7, 147.6, 136.9, 136.8, 130.7, 130.3, 130.0, 128.7, 128.7, 128.1, 128.0, 127.5, 127.2, 127.2, 126.9, 124.4, 123.7, 123.1, 114.7, 114.1, 104.2, 103.7, 71.2, 71.0, 60.8, 56.7 ppm. **ESI-MS**: m/z calcd. for $C_{16}H_{15}ClO_2H^+$ [M+H$^+$]: 275.1, found: 275.1.

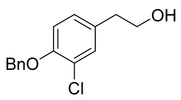

2-(4-(Benzyloxy)-3-chlorophenyl)ethan-1-ol (17(Cl)) To a solution of 1-(benzyl-oxy)-2-chloro-4-(2-methoxyvinyl)benzene (3.60 g, 13.1 mmol, 1.0 eq.) in THF (80 mL) and H_2O (150 mL) $Hg(OAc)_2$ (4.59 g, 4.4 mmol, 1.1 eq.) was added at 0 °C. The reaction solution was stirred for 10 min at 0 °C before a saturated K_2CO_3 solution (80 mL) was added slowly, followed by portion wise addition of $NaBH_4$ (1.98 g, 52.4 mmol, 4.0 eq.) at 0°C. The reaction mixture was stirred for 30 min at 0 °C. Afterwards the mixture was transferred into a separation flask and the aqueous layer extracted with EtOAc (4 x 150 mL). The combined organic layer was washed with saturated NaCl-solution (250 mL), dried over Na_2SO_4 and concentrated *in vacuo*. The product was isolated over column chromatography (PE:EtOAc = 1:1) as a colourless oil (1.20 g, 4.6 mmol, 35 % (o2s)).
^{1}H NMR (400 MHz, $CDCl_3$): δ = 7.55 (d, *J* = 7.5 Hz, 2H), 7.47 (t, *J* = 7.5 Hz, 2H), 7.41 (t, *J* = 7.2 Hz, 1H), 7.35 (dd, *J* = 3.6, 1.2 Hz, 1H), 7.12 (dd, *J* = 8.4, 1.7 Hz, 1H), 6.99 (d, *J* = 8.3 Hz, 1H), 5.23 (s, 2H), 3.90 (t, *J* = 6.3 Hz, 2H), 2.86 (t, *J* = 6.5 Hz, 2H). **^{13}C NMR** (101 MHz, $CDCl_3$): δ = 153.0, 136.8, 132.3, 130.9, 128.7, 128.3, 128.1, 127.2, 123.4, 114.4, 71.1, 63.6, 38.1 ppm. **ESI-MS**: m/z calcd. for $C_{15}H_{15}ClO_2Na^+$ [M+Na$^+$]: 285.1, found: 285.1.

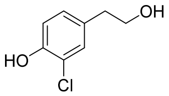

2-Chloro-4-(2-hydroxyethyl)phenol (18(Cl)) To a solution of 2-(4-(benzyloxy)-3-chlorophenyl)ethan-1-ol (1.20 g, 4.57 mmol, 1.0 eq.) in MeOH (20 mL) Pd/C (83 mg, 0.78 mmol, 0.17 eq.) was added. The suspension was stirred under H_2-atmosphere for 3 h. Afterwards the mixture was filtered over Celite. The solvent was evaporated under reduced to afford the title compound as a colourless oil (787 mg, 4.57 mmol, quant.).
^{1}H NMR (400 MHz, $CDCl_3$): δ = 7.16 (d, *J* = 1.9 Hz, 1H), 6.98 (dd, *J* = 8.3, 1.9 Hz, 1H), 6.90 (d, *J* = 8.2 Hz, 1H), 3.78 (dd, *J* = 7.4, 5.7 Hz, 2H), 2.74 (t, *J* = 6.4 Hz, 2H) ppm. **^{13}C NMR** (101 MHz, $CDCl_3$): δ = 150.6, 131.5, 129.7, 128.8, 120.1, 116.6, 63.5, 38.1 ppm. **ESI-MS**: m/z calcd. for $C_8H_9ClO_2Na^+$ [M+Na$^+$]: 195.0, found: 195.1.

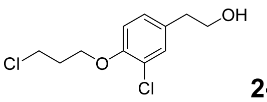

2-(3-Chloro-4-(3-chloropropoxy)phenyl)ethan-1-ol (19(Cl)) To a suspen-sion of 2-chloro-4-(2-hydroxyethyl)phenol (760 mg, 4.4 mmol, 1.0 eq.) and Cs_2CO_3 (2.87 g, 8.8

mmol, 2.0 eq.) in DMF (20 mL) was added a solution of 1-chloro-3-iodopropane (710 µL, 6.6 mmol, 1.5 eq.) in DMF (3 mL) and the suspension was stirred at 70 °C for 16 h. After cooling to ambient temperature, the reaction mixture was diluted with water (50 mL) and extracted with EtOAc (3 x 30 mL). The combined organic layer was washed with saturated NaCl-solution (50 mL), dried over Na_2SO_4 and concentrated *in vacuo*. The product was isolated by column chromatography (PE:EtOAc = 2:1) as a yellow oil (587 mg, 2.4 mmol, 54 %).

^{1}H NMR (400 MHz, $CDCl_3$): δ = 7.24 (d, *J* = 2.1 Hz, 1H), 7.06 (td, *J* = 8.2, 2.1 Hz, 1H), 6.89 (d, *J* = 8.4 Hz, 1H), 4.16 (t, *J* = 5.8 Hz, 2H), 3.81 (dt, *J* = 8.5, 6.4 Hz, 4H), 2.78 (t, *J* = 6.5 Hz, 2H), 2.32 – 2.24 (m, 2H) ppm. **^{13}C NMR** (101 MHz, $CDCl_3$): δ = 153.0, 132.3, 130.8, 128.4, 123.2, 113.9, 65.7, 63.6, 41.6, 38.1, 32.6 ppm. **ESI-MS**: m/z calcd. for $C_{11}H_{14}Cl_2O_2Na^+$ [M+Na^+]: 271.0, found: 271.0.

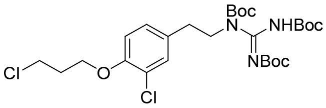

1-(3-Chloro-4-(3-chloropropoxy)phenethyl)-*N,N',N''*-tri-Boc-guanidine (20(Cl)) A suspension of 2-(3-chloro-4-(3-chloropropoxy)phenyl)ethan-1-ol (350 mg, 1.4 mmol, 1.0 eq.), PPh_3 (553 mg, 2.1 mmol, 1.5 eq.) and *N,N',N''*-tri-Boc-guanidine (758 mg, 2.1 mmol, 1.5 eq.) in dry THF (15 mL) was cooled to 0 °C before a premixed solution of DIAD (414 µL, 2.1 mmol, 1.5 eq.) in THF (1 mL) was added. The solution was warmed to r.t. and stirred for 20 h. After removal of the solvent *in vacuo* the residue was dissolved in water (30 mL) and extracted with EtOAc (3 x 30 mL). The combined organic layer was washed with saturated NaCl-solution (50 mL) and dried over anhydrous Na_2SO_4. The product was isolated by column chromatography (PE:EtOAc = 5:1) as a colorless oil (530 mg, 0.9 mmol, 64 %).

^{1}H NMR (400 MHz, $CDCl_3$): δ = 7.24 (s, 1H), 7.06 (dt, *J* = 6.9, 3.5 Hz, 1H), 6.84 (d, *J* = 8.5 Hz, 1H), 4.13 (t, *J* = 6.4 Hz, 2H), 3.99 – 3.94 (m, 2H), 3.79 (t, *J* = 6.3 Hz, 2H), 2.93 – 2.86 (m, 2H), 2.30 – 2.23 (m, 2H), 1.51 (s, 18H), 1.46 (s, 9H) ppm. **^{13}C NMR** (101 MHz, $CDCl_3$): δ = 153.5, 152.9, 152.1, 132.8, 130.9, 128.4, 123.0, 113.8, 83.6, 65.7, 49.1, 41.6, 34.1, 32.4, 28.2, 28.1 ppm. **ESI-MS**: m/z calcd. for $C_{27}H_{41}Cl_2N_3O_7H^+$ [M+H^+]: 590.2, found: 590.4.

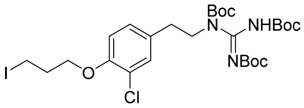

1-(3-Chloro-4-(3-iodopropoxy)phenethyl)- *N,N',N''*-tri-Boc -guanidine (21(Cl)) To a solution of compound 20(Cl) (250 mg, 0.4 mmol, 1.0 eq.) in dry acetone (10 mL) NaI (634 mg, 4.2 mmol, 10.0 eq.) was added. The solution was stirred at 70 °C for 18 h. More equivalents of NaI were added until a full conversion after 3 days. The solution was diluted with water (10 mL) and extracted with Et_2O (4 x 20 mL). The combined organic layer was washed with saturated NaCl-solution (50 mL), dried over anhydrous Na_2SO_4, filtered and concentrated *in vacuo*. The title compound was isolated by column chromatography (PE:EtOAc = 10:1) as a colourless oil (113 mg, 0.17 mmol, 39 %).

^{1}H NMR (400 MHz, $CDCl_3$): δ = 7.25 (s, 1H), 7.06 (td, *J* = 8.3, 1.9 Hz, 1H), 6.84 (d, *J* = 8.4 Hz, 1H), 4.07 (t, *J* = 5.7 Hz, 2H), 3.99 – 3.95 (m, 2H), 3.42 (t, *J* = 6.7 Hz, 2H), 2.91 – 2.86 (m, 2H), 2.29 (dd, *J* = 12.2, 6.0 Hz, 2H), 1.51 (s, 18H), 1.46 (s, 9H) ppm. **^{13}C NMR** (101 MHz, $CDCl_3$): δ = 153.5, 152.8, 132.9, 131.0, 128.4, 123.1, 113.9, 83.6, 68.7, 49.1, 34.2, 33.1, 28.3, 28.1, 2.7 ppm. **ESI-MS**: m/z calcd. for $C_{27}H_{41}ClIN_3O_7H^+$ [M+H^+]: 682.2, found: 682.2.

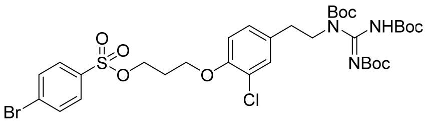

(*E*)-3-(2-Chloro-4-(2-(1,2,3-tris(tert-butoxycarbonyl)-guanidino)ethyl)phenoxy)propyl 4-bro-mobenzenesulfonate (22(Cl)) The starting 1-(3-chloro-4-(3-iodopropoxy)phenethyl)- *N,N',N"*-tri-Boc-guanidine (95 mg, 0.14 mmol, 1.0 eq.) was dissolved in dry MeCN (15 mL). Silver(I) 4-bromobenzenesulfonate (239 mg, 0.70 mmol, 5.0 eq.) was added at 0 °C and the solution was stirred at r.t. for 7 d. The solution was then diluted with water (20 mL) and extracted with EtOAc (3 x 30 mL). The combined organic layer was washed with saturated NaCl solution (75 mL) and dried over anhydrous Na_2SO_4, filtered and concentrated *in vacuo*. The title compound was isolated by column chromatography (PE:EtOAc = 7:1) as a white solid (82 mg, 0.10 mmol, 75 %.)

^{1}H NMR (400 MHz, $CDCl_3$): δ = 10.56 (s, 1H), 7.69 (d, *J* = 8.2 Hz, 2H), 7.51 (d, *J* = 8.2 Hz, 2H), 7.24 (d, *J* = 1.7 Hz, 1H), 7.04 (dd, *J* = 8.3, 1.7 Hz, 1H), 6.68 (d, *J* = 8.3 Hz, 1H), 4.32 (t, *J* = 5.8 Hz, 2H), 4.01 – 3.92 (m, 4H), 2.94 – 2.86 (m, 2H), 2.19 – 2.12 (m, 2H), 1.51 (s, 18H), 1.47 (s, 9H) ppm. **^{13}C NMR** (101 MHz, $CDCl_3$): δ = 153.5, 152.4, 152.2, 134.8, 132.8, 132.6, 131.0, 129.3, 129.1, 128.3, 122.7, 113.1, 83.6, 67.4, 63.9, 49.1, 34.1, 28.9, 28.3, 28.1 ppm. **ESI-MS**: m/z calcd. for $C_{33}H_{45}BrClN_3O_{10}SH^+$ [M+H$^+$]: 790.2, found: 790.2.

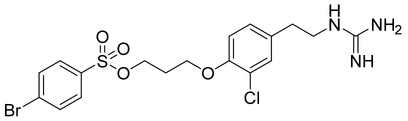

3-(2-Chloro-4-(2-guanidinoethyl)phenoxy)propyl 4-bromo-benzenesulfonate (23) Compound 22(Cl) (10 mg, 12.6 µmol) was dissolved in dry CH_2Cl_2 (375 µL). At 0 °C TFA (125 µL) was added and the solution was stirred at r.t. for 18 h. Afterwards, the solvent was evaporated and the target compound was obtained as the corresponding TFA salt (6 mg, 9.9 µmol, 79 %).

^{1}H NMR (400 MHz, MeOD): δ = 7.80 – 7.75 (m, 2H), 7.67 – 7.63 (m, 2H), 7.31 (d, *J* = 2.1 Hz, 1H), 7.17 (dd, *J* = 8.4, 2.1 Hz, 1H), 6.93 (d, *J* = 8.4 Hz, 1H), 4.35 (t, *J* = 5.8 Hz, 2H), 4.01 (t, *J* = 5.7 Hz, 2H), 3.47 (t, *J* = 7.1 Hz, 2H), 2.87 (t, *J* = 7.1 Hz, 2H), 2.21 – 2.13 (m, 2H) ppm. **ESI-MS**: m/z calcd. for $C_{18}H_{21}BrClN_3O_4SH^+$ [M+H$^+$]: 490.0, found: 490.0

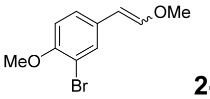

2-Bromo-1-methoxy-4-(2-methoxyvinyl)benzene (16(Br)) To a suspension of (methoxymethyl) triphenylphosphonium chloride (11.96 g, 34.9 mmol, 1.5 eq.) in dry THF (100 mL) under argon atmosphere and 0 °C t-BuOK (5.22 g, 46.5 mmol, 2.0 eq.) was added and the red reaction mixture was stirred for 2 min. Afterwards a solution of 3-bromo-4-methoxy benzaldehyde (5.00 g, 23.3 mmol, 1.0 eq.) in dry THF (20 mL) was added and the dark red reaction solution was stirred at r.t. for 18 h. Afterwards the solvent was removed *in vacuo* and the residue was portioned between water (50 mL) and EtOAc (50 mL) and the aqueous layer was extracted with EtOAc (2 x 50 mL). The combined organic layer was washed with saturated NaCl-solution (100 mL), dried over Na_2SO_4 and concentrated under reduced pressure. The product was isolated over column chromatography (PE:EtOAc = 4:1) as a colourless oil (5.48 g, 19.9 mmol, 86 %).

^{1}H NMR (400 MHz, $CDCl_3$): δ = 7.82 (d, *J* = 2.1 Hz, 1H), 7.43 (dt, *J* = 4.3, 2.1 Hz, 2H), 7.11 (dd, *J* = 8.5, 2.2 Hz, 1H), 6.92 (d, *J* = 13.0 Hz, 1H), 6.81 (dd, *J* = 8.5, 5.4 Hz, 2H), 6.08 (d, *J* = 7.0 Hz, 1H), 5.71 (d, *J* = 13.0 Hz, 1H), 5.11 (d, *J* = 7.0 Hz, 1H), 3.87 (d, *J* = 2.6 Hz, 6H), 3.77 (s, 3H), 3.66 (s, 3H) ppm. **^{13}C NMR** (101 MHz, $CDCl_3$): δ = 154.1, 153.8, 148.6, 147.5, 133.0, 130.7, 130.4,

129.9, 128.4, 125.3, 112.3, 112.0, 111.7, 111.5, 104.1, 103.6, 60.8, 56.7, 56.5, 56.4 ppm. **ESI-MS**: m/z calcd. for $C_{10}H_{11}BrO_2H^+$ [M+H$^+$]: 243.0, found: 243.0.

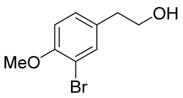

2-(3-Bromo-4-methoxyphenyl)ethan-1-ol (17(Br)) To a solution of 2-bromo-1-methoxy-4-(2-methoxyvinyl)benzene (2.50 g, 10.3 mmol, 1.0 eq.) in THF (60 mL) and H_2O (100 mL) $Hg(OAc)_2$ (3.60 g, 11.3 mmol, 1.1 eq.) was added at 0 °C. The reaction solution was stirred for 10 min at 0 °C before a saturated K_2CO_3 solution (50 mL) was added slowly, followed by portion wise addition of $NaBH_4$ (1.56 g, 41.1 mmol, 4.0 eq.) at 0°C. The reaction mixture was stirred for 30 min at 0 °C. Afterwards the mixture was transferred into a separation flask and the aqueous layer extracted with EtOAc (4 x 100 mL). The combined organic layer was washed with saturated NaCl-solution (200 mL), dried over Na_2SO_4 and concentrated *in vacuo*. The product was isolated over column chromatography (PE:EtOAc = 1:1) as a colourless oil (2.38 g, 5.9 mmol, 57 %).
^{1}H NMR (400 MHz, $CDCl_3$): δ = 7.42 (d, *J* = 2.1 Hz, 1H), 7.13 (dd, *J* = 8.3, 2.1 Hz, 1H), 6.84 (d, *J* = 8.4 Hz, 1H), 3.87 (s, 3H), 3.82 (s, 2H), 2.78 (t, *J* = 6.5 Hz, 2H) ppm. **^{13}C NMR** (101 MHz, $CDCl_3$): δ = 154.7, 133.8, 132.3, 129.1, 112.2, 111.8, 63.7, 56.4, 38.0 ppm. **ESI-MS**: m/z calcd. for $C_9H_{11}BrO_2Na^+$ [M+Na$^+$]: 253.0, found: 253.0

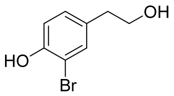

2-Bromo-4-(2-hydroxyethyl)phenol (18(Br)) A solution of 2-(3-bromo-4-methoxyphenyl)ethan-1-ol (1.0 g, 4.33 mmol, 1.0 eq.) in dry CH_2Cl_2 (30 mL) under Argon was cooled to – 20 °C before BBr_3 (3.0 mL, 3.00 mmol, 0.7 eq., 1 M solution in CH_2Cl_2) was added slowly. The solution was stirred for 5 min at – 20 °C. Afterwards it was diluted with water (10 mL) and proportionate between saturated $NaHCO_3$-solution (30 mL) and EtOAc (30 mL). The aqueous phase was extracted with EtOAc (3 x 30 mL). the combined organic layer was washed with saturated NaCl-solution (70 mL), dried over anhydrous Na_2SO_4, filtered and concentrated *in vacuo.* The title compound was isolated by column chromatography (PE:EtOAc = 2:1) as a colourless oil (811 mg, 3.73 mmol, 86 %).
^{1}H NMR (400 MHz, $CDCl_3$): δ = 7.34 (d, *J* = 2.0 Hz, 1H), 7.07 (dd, *J* = 8.3, 2.0 Hz, 1H), 6.95 (d, *J* = 8.3 Hz, 1H), 5.54 (s, 1H), 3.82 (t, *J* = 6.5 Hz, 2H), 2.78 (t, *J* = 6.5 Hz, 2H) ppm. **^{13}C NMR** (101 MHz, $CDCl_3$): δ = 151.1, 132.4, 132.3, 129.9, 116.3, 110.3, 63.7, 38.0 ppm. **ESI-MS**: m/z calcd. for $C_8H_9BrO_2Na^+$ [M+Na$^+$]: 239.0, found: 239.0.

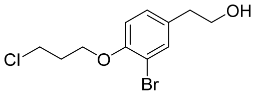

2-(3-Bromo-4-(3-chloropropoxy)phenyl)ethan-1-ol (19(Br)) To a suspension of 2-bromo-4-(2-hydroxyethyl)phenol (500 mg, 2.3 mmol, 1.0 eq.) and Cs_2CO_3 (1.1 g, 3.5 mmol, 1.5 eq.) in DMF (15 mL) was added a solution of 1-chloro-3-iodopropane (370 µL, 3.5 mmol, 1.5 eq.) in DMF (1 mL) and the suspension was stirred at 70 °C for 16 h. After cooling to ambient temperature, the reaction mixture was diluted with water (30 mL) and extracted with EtOAc (3 x 30 mL). The combined organic layer was washed with saturated NaCl-solution (50 mL), dried over Na_2SO_4 and concentrated *in vacuo*. The product was isolated by column chromatography (PE:EtOAc = 2:1) as a yellow oil (270 mg, 0.9 mmol, 40 %).
^{1}H NMR (400 MHz, $CDCl_3$): δ = 7.41 (d, *J* = 2.0 Hz, 1H), 7.11 (dd, *J* = 8.3, 2.1 Hz, 1H), 6.88 – 6.84 (m, 1H), 4.15 (t, *J* = 5.7 Hz, 2H), 3.81 (td, *J* = 6.3, 3.0 Hz, 4H), 2.78 (t, *J* = 6.4 Hz, 2H), 2.32 – 2.22 (m, 2H) ppm. **^{13}C NMR** (101 MHz, $CDCl_3$): δ = 153.8, 133.8, 132.8, 129.1, 113.6, 112.5, 65.7, 63.6, 41.6, 38.1, 32.3 ppm. **ESI-MS**: m/z calcd. for $C_{11}H_{14}BrClO_2Na^+$ [M+Na$^+$]: 315.0, found: 315.0.

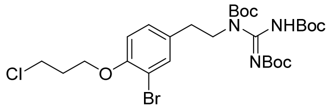

1-(3-Bromo-4-(3-chloropropoxy)phenethyl)-*N,N',N"*-tri-Boc-guanidine (20(Br)) A suspension of 2-(3-bromo-4-(3-chloropropoxy)phenyl)ethan-1-ol (300 mg, 1.0 mmol, 1.0 eq.), PPh_3 (402 mg, 1.5 mmol, 1.5 eq.) and *N,N',N"*-tri-Boc-guanidine (550 mg, 2.1 mmol, 1.5 eq.) in dry THF (15 mL) was cooled to 0 °C before a premixed solution of DIAD (300 µL, 1.5 mmol, 1.5 eq.) in THF (1 mL) was added. The solution was warmed to r.t. and stirred for 20 h. After removal of the solvent *in vacuo* the residue was dissolved in water (30 mL) and extracted with EtOAc (3 x 30 mL). The combined organic layer was washed with saturated NaCl-solution (50 mL) and dried over anhydrous Na_2SO_4. The product was isolated by column chromatography (PE:EtOAc = 6:1) as a colorless oil (300 mg, 0.47 mmol, 46 %).

^{1}H NMR (400 MHz, $CDCl_3$): δ = 7.43 (d, *J* = 2.0 Hz, 1H), 7.11 (dd, *J* = 8.3, 2.1 Hz, 1H), 6.81 (d, *J* = 8.4 Hz, 1H), 4.16 – 4.11 (m, 2H), 3.96 (dd, *J* = 8.5, 6.7 Hz, 2H), 3.80 (t, *J* = 6.3 Hz, 2H), 2.88 (dd, *J* = 9.3, 5.9 Hz, 2H), 2.30 – 2.22 (m, 2H), 1.51 (s, 18H), 1.46 (s, 9H) ppm. **^{13}C NMR** (101 MHz, $CDCl_3$): δ = 153.7, 153.5, 152.1, 133.9, 133.3, 129.2, 113.5, 112.3, 83.6, 65.7, 49.1, 41.7, 34.0, 32.4, 28.3, 28.1 ppm. **ESI-MS**: m/z calcd. for $C_{27}H_{41}BrClN_3O_7H^+$ [M+H$^+$]: 634.2, found: 634.2.

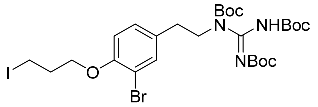

1-(3-Bromo-4-(3-iodopropoxy)phenethyl)-*N,N',N"*-tri-Boc-guanidine (21(Br)) To a solution of the previous step (275 mg, 0.4 mmol, 1.0 eq.) in dry acetone (10 mL) NaI (650 mg, 4.3 mmol, 10.0 eq.) was added. The solution was stirred at 70 °C for 18 h. More equivalents of NaI were added until a full conversion after 3 days. The solution was diluted with water (10 mL) and extracted with Et_2O (4 x 20 mL). The combined organic layer was washed with saturated NaCl-solution (50 mL), dried over anhydrous Na_2SO_4, filtered and concentrated *in vacuo*. The title compound was isolated by column chromatography (PE:EtOAc = 8:1) as a colourless oil (111 mg, 0.15 mmol, 35 %).

^{1}H NMR (400 MHz, $CDCl_3$): δ = 7.43 (d, *J* = 2.0 Hz, 1H), 7.11 (dd, *J* = 8.3, 1.9 Hz, 1H), 6.82 (d, *J* = 8.3 Hz, 1H), 4.06 (t, *J* = 5.7 Hz, 2H), 3.99 – 3.94 (m, 2H), 2.93 – 2.85 (m, 2H), 2.34 – 2.25 (m, 2H), 1.51 (s, 18H), 1.46 (s, 9H) ppm. **^{13}C NMR** (101 MHz, $CDCl_3$): δ = 153.7, 153.5, 134.0, 133.3, 129.2, 113.6, 112.4, 83.6, 68.7, 49.1, 34.1, 33.0, 28.3, 28.1, 2.8 ppm. **ESI-MS**: m/z calcd. for $C_{27}H_{41}BrIN_3O_7H^+$ [M+H$^+$]: 726.1, found: 726.2.

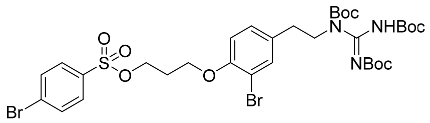

(*E*)-3-(2-Bromo-4-(2-(1,2,3-tris(tert-butoxycarbonyl)-guanidino)ethyl)phenoxy)propyl 4-bromobenzenesulfonate (22(Br)) The starting 1-(3-bromo-4-(3-iodopropoxy)phenethyl)- *N,N',N"*-tri-Boc-guanidine (80 mg, 0.11 mmol, 1.0 eq.) was dissolved in dry MeCN (15 mL). Silver(I) 4-bromobenzenesulfonate (189 mg, 0.55 mmol, 5.0 eq.) was added at 0 °C and the solution was stirred at r.t. for 7 d. The solution was then diluted with water (20 mL) and extracted with EtOAc (3 x 30 mL). The combined organic layer was washed with saturated NaCl solution (75 mL) and dried over anhydrous Na_2SO_4, filtered and concentrated *in vacuo*. The title compound was isolated by column chromatography (PE:EtOAc = 7:1) as a white solid (77 mg, 0.09 mmol, 84 %.)

¹H NMR (400 MHz, $CDCl_3$): δ = 10.56 (s, 1H), 7.69 (d, *J* = 8.3 Hz, 2H), 7.50 (d, *J* = 8.3 Hz, 2H), 7.41 (d, *J* = 1.6 Hz, 1H), 7.09 (dd, *J* = 8.3, 1.5 Hz, 1H), 6.65 (d, *J* = 8.3 Hz, 1H), 4.34 (t, *J* = 5.8 Hz, 2H), 3.95 (dt, *J* = 11.3, 6.6 Hz, 4H), 2.93 – 2.87 (m, 2H), 2.16 (p, *J* = 5.7 Hz, 2H), 1.51 (s, 18H), 1.47 (s, 9H) ppm. **¹³C NMR** (101 MHz, $CDCl_3$): δ = 153.5, 153.2, 152.1, 134.8, 134.0, 133.3, 132.7, 129.3, 129.1, 129.1, 112.8, 112.1, 83.6, 67.5, 63.9, 49.1, 34.0, 28.9, 28.3, 28.1 ppm. **ESI-MS**: m/z calcd. for $C_{33}H_{45}Br_2N_3O_{10}SH^+$ [M+H⁺]: 834.1, found: 834.2.

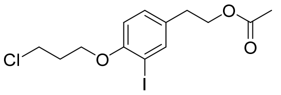

4-(3-Chloropropoxy)-3-iodophenethyl acetate (23) 4-Hydroxy-3-iodophenethyl acetate (330 mg, 1.08 mmol, 1.0 eq.) was dissolved in acetone (17 mL) followed by the addition of 3-chloro-1-iodopropane (177 µL, 1.65 mmol, 1.5 eq.) and Cs_2CO_3 (702 mg, 2.15 mmol, 2.0 eq.). The reaction mixture was stirred at 70 °C for 18 h before water (85 mL) was added. After an extraction with EtOAc (3 × 80 mL), the combined organic layer was washed with saturated NaCl-solution (200 mL) and dried over Na_2SO_4. The solvent was removed under reduced pressure and the crude product was purified by column chromatography (PE:EtOAc = 5:1) to give the title compound as a yellow oil (372 mg, 0.97 mmol, 90 % yield).
¹H NMR ($CDCl_3$, 400 MHz): δ = 7.63 (1H, d, *J* = 2.16 Hz), 7.13 (1H, dd, *J* = 2.15 Hz, 8.33 Hz), 6.76 (1H, d, *J* = 8.35 Hz), 4.22 (2H, t, *J* = 6.99 Hz), 4.14 (2H, t, *J* = 5.68 Hz), 3.84 (2H, t, *J* = 6.33 Hz), 2.83 (2H, t, *J* = 6.99 Hz), 2.23 (2H, quint, *J* = 6.00 Hz), 2.03 (3H, s) ppm. **¹³C NMR** ($CDCl_3$, 101 MHz): δ = 171.1, 156.1, 139.8, 132.5, 130.0, 112.2, 86.8, 65.6, 64.9, 41.8, 33.8, 32.3, 21.1 ppm. **ESI-MS**: m/z calcd. for $C_{13}H_{16}O_3IClNa^+$ [M+Na⁺]: 405.0, found: 404.8.

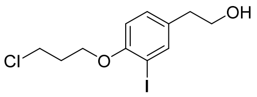

2-(4-(3-Chloropropoxy)-3-iodophenyl)ethan-1-ol (19(I)) 4-(3-Chloro-propoxy)-3-iodophenethyl acetate (189 mg, 0.49 mmol, 1.0 eq.) was dissolved in MeOH (9 mL) before K_2CO_3 (650 mg, 4.70 mmol, 9.6 eq.) was added. The reaction mixture was stirred at room temperature for 3 h and was partitioned between water (60 mL) and CH_2Cl_2 (60 mL) followed by layer separation. The aqueous layer was extracted with CH_2Cl_2 (2 × 60 mL) and the combined organic layer was washed with saturated NaCl-solution (180 mL) and dried over Na_2SO_4. The solvent was removed under reduced pressure to obtain the alcohol as a yellow oil (175 mg, quant.) which was used directly in the next step.
¹H NMR ($CDCl_3$, 400 MHz): δ = 7.65 (1H, d, *J* = 2.15 Hz), 7.16 (1H, dd, *J* = 2.12 Hz, 8.32 Hz), 6.77 (1H, d, *J* = 8.36 Hz), 4.14 (2H, t, *J* = 5.72 Hz), 3.87 – 3.78 (4H, m), 2.77 (2H, t, *J* = 6.48 Hz, 2.27 (2H, quint, *J* = 6.00 Hz) ppm. **¹³C NMR** ($CDCl_3$, 101 MHz): δ = 156.1, 139.9, 133.3, 130.2, 112.3, 87.0, 65.7, 63.7, 41.8, 37.9, 32.3 ppm. **ESI-MS**: m/z calcd. for $C_{11}H_{14}O_2IClNa^+$ [M+Na⁺]: 363.0, found: 362.8.

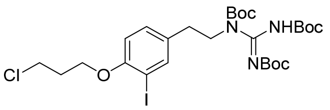

1-(4-(3-Chloropropoxy)-3-iodophenethyl)-*N*,*N'*,*N''*-tri-Boc-guanidine (20(I)) 2-(4-(3-Chloropropoxy)-3-iodophenyl)ethan-1-ol (182 mg, 0.53 mmol, 1.0 eq.), PPh_3 (210 mg, 0.80 mmol, 1.5 eq.) and *N*,*N'*,*N''*-tri-Boc-guanidine (288 mg, 0.70 mmol, 1.3 eq.) were dissolved in dry THF (15 mL) under inert gas atmosphere. DIAD (158 µL, 0.80 mmol, 1.5 eq.) was added at 0 °C and the reaction mixture was stirred at room temperature for 18 h. The solvent was removed under reduced pressure and the residue was partitioned between water (20 mL) and CH_2Cl_2 (20 mL) followed by layer separation. The aqueous layer was extracted with CH_2Cl_2 (2 × 20

mL) and the combined organic layer was washed with saturated NaCl-solution (60 mL) and dried over Na_2SO_4. Evaporation of the solvent under reduced pressure and purification by column chromatography (PE:EtOAc = 5:1) afforded the title compound as a colourless oil (234 mg, 0.34 mmol, 64% yield).

^{1}H NMR ($CDCl_3$, 400 MHz): δ = 10.59 (1H, bs), 7.65 (1H, d, *J* = 2.13 Hz), 7.15 (1H, dd, *J* = 2.13 Hz, 8.36 Hz), 6.73 (1H, d, *J* = 8.40 Hz), 4.12 (2H, t, *J* = 70 Hz), 3.99 - 3.92 (2H, m), 3.83 (2H, t, *J* = 6.31 Hz), 2.92 – 2.83 (2H, m), 2.26 (2H, quint, *J* = 6.00 Hz), 1.51 (18 H, s), 1.46 (9H, s) ppm. **^{13}C NMR** ($CDCl_3$, 101 MHz): δ = 156.0, 153.6/152.0, 140.0, 133.8, 130.3, 112.2, 86.8, 83.6, 65.7, 49.1, 41.8, 33.9, 32.4, 28.3/28.1 ppm. **ESI-MS**: m/z calcd. for $C_{27}H_{41}N_3O_7IClH^+$ [M+H$^+$]: 682.2, found: 682.1.

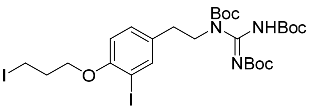

1-(4-(3-Iodopropoxy)-3-iodophenethyl)-*N*,*N'*,*N''*-tri-Boc-guanidine (21(I)) 1-(4-(3-Chloropropoxy)-3-iodophenethyl)-*N*,*N'*,*N''*-tri-Boc-guanidine (205 mg, 0.30 mmol, 1.0 eq.) was dissolved in acetone (10 mL) and NaI (270 mg, 1.80 mmol, 6.0 eq.) was added. The reaction mixture was stirred at 70 °C for 18 h after which time LC-MS still indicated the presence of unreacted starting material. Therefore, an additional amount of NaI (451 mg, 3.01 mmol, 10.0 eq.) was added and the reaction mixture was stirred at 70 °C for another day. Another amount of NaI (451 mg, 3.01 mmol, 10.0 eq.) was added and the reaction mixture was stirred at 70 °C for additional 18 h. Water (15 mL) was added and the mixture was extracted with Et_2O (4 × 15 mL). The combined organic layer was washed with saturated NaCl-solution (30 mL) and dried over Na_2SO_4. The solvent was removed under reduced pressure to give the iodide as an orange oil (179 mg, 0.23 mmol, 77% yield) which was used directly in the next step.

^{1}H NMR ($CDCl_3$, 400 MHz): δ = 10.59 (1H, s), 7.65 (1H, d, *J* = 2.14 Hz), 7.15 (1H, dd, *J* = 2.08 Hz, 8.31 Hz), 6.73 (1H, d, *J* = 8.39 Hz), 4.05 (3H, t, *J* = 5.63 Hz), 3.99 - 3.91 (2H, m), 3.46 (3H, t, *J* = 6.61 Hz), 2.91 – 2.83 (2H, m), 2.32 - 2.25 (3H, m), 1.54 – 1.48 (19 H, m), 1.46 (9H, s) ppm. **^{13}C NMR** ($CDCl_3$, 101 MHz): δ = 155.8, 153.6/151.9, 140.0, 133.9, 130.2, 112.2, 86.8, 83.6, 68.6, 49.1, 33.8, 33.0, 28.3/28.1, 3.1 ppm. **ESI-MS**: m/z calcd. for $C_{27}H_{41}N_3O_7I_2H^+$ [M+H$^+$]: 774.1, found: 774.1.

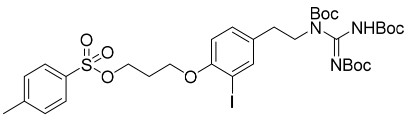

3-(2-Iodo-4-(2-(1,2,3-tris(*tert*-butoxycarbonyl)-guanidino)-ethyl)phenoxy)propyl 4-methylbenzenesulfonate (24) 1-(4-(3-Iodopropoxy)-3-iodophenethyl)-*N*,*N'*,*N''*-tri-Boc-guanidine (144 mg, 0.19 mmol, 1.0 eq.) was dissolved in MeCN (18 mL) and silver *p*-toluenesulfonate (520 mg, 1.86 mmol, 9.8 eq.) was added. The reaction mixture was stirred in darkness at room temperature for 5 days and partitioned between water (15 mL) and EtOAc (15 mL) followed by layer separation. The aqueous layer was extracted with EtOAc (2 × 15 mL) and the combined organic layer was washed with saturated NaCl-solution (40 mL) and dried over Na_2SO_4. The solvent was removed under reduced pressure and the crude material was purified by column chromatography (PE:EtOAc = 4:1) to obtain the target compound as a pale green oil (83 mg, 0.10 mmol, 55% yield).

^{1}H NMR ($CDCl_3$, 400 MHz): δ = 10.52 (1H, bs), 7.74 (2H, d, *J* = 8.35 Hz), 7.61 (1H, d, *J* = 2.05 Hz), 7.20 (2H, d, *J* = 8.26 Hz), 7.12 (1H, dd, *J* = 2.12 Hz, 8.36 Hz), 6.58 (1H, d, *J* = 8.37 Hz), 4.31 (2H, t, *J* = 6.00 Hz), 3.98 – 3.89 (4H, m), 2.91 - 2.83 (2H, m), 2.35 (3H, s), 2.18 – 2.10 (3H, m), 1.51 (18 H, s), 1.47 (9H, s) ppm. **^{13}C NMR** ($CDCl_3$, 101 MHz): δ = 155.6, 153.5, 144.9, 139.8, 133.7, 132.8, 130.1, 130.0, 128.0, 111.8, 86.7, 83.6, 67.3, 64.3, 49.2, 33.9, 29.0, 28.3/28.1, 21.8 ppm. **ESI-MS**: m/z calcd. for $C_{34}H_{48}N_3O_{10}SIH^+$ [M+H$^+$]: 818.2, found: 818.1.

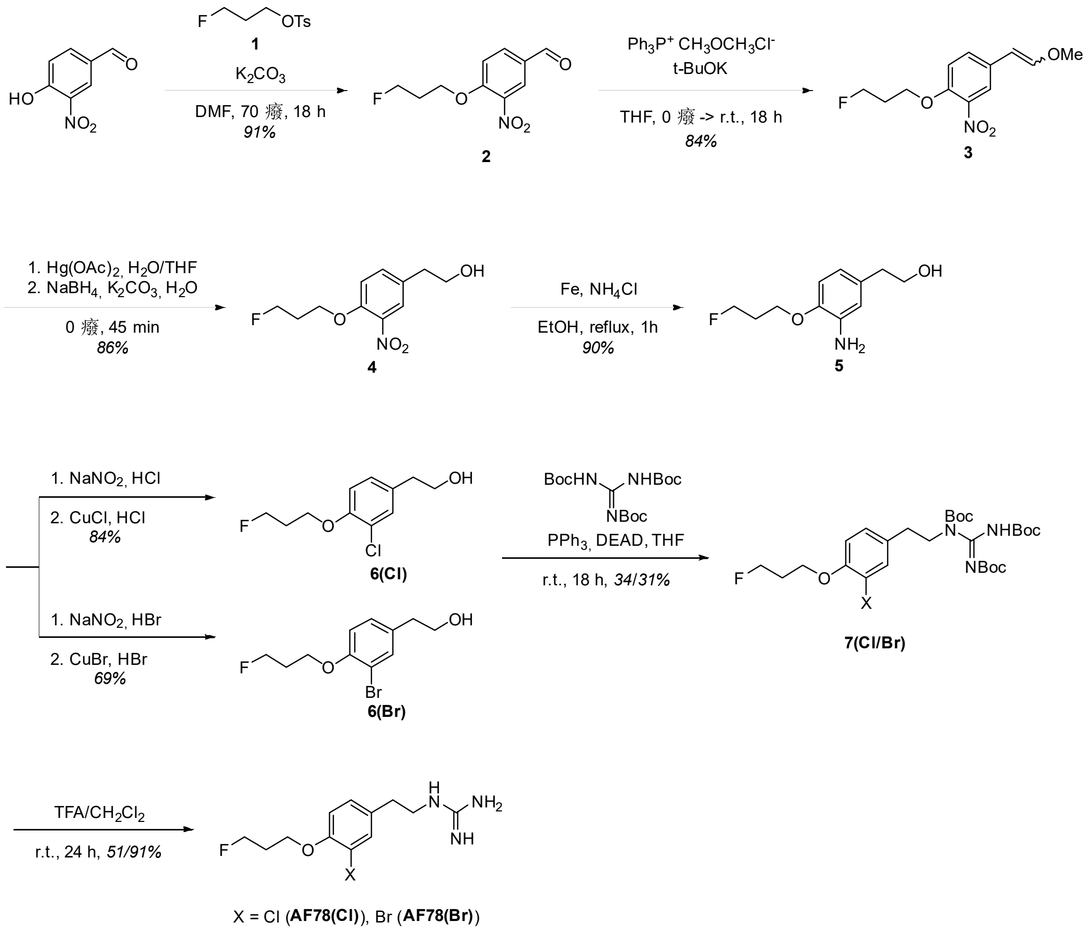

Figure S1. Synthesis of cold derivatives AF78(Cl) & AF78(Br).

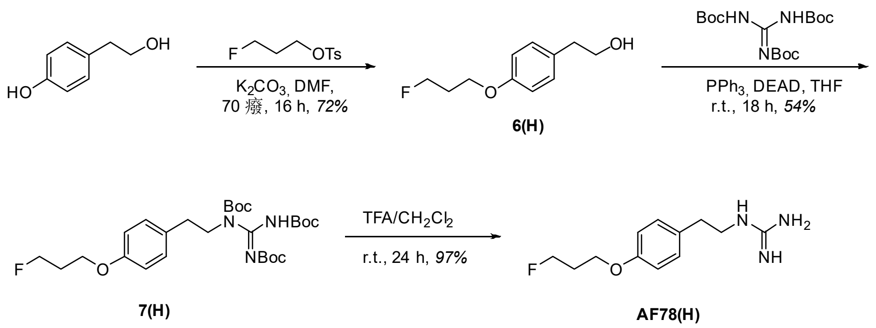

Figure S2. Synthesis of cold derivative AF78(H).

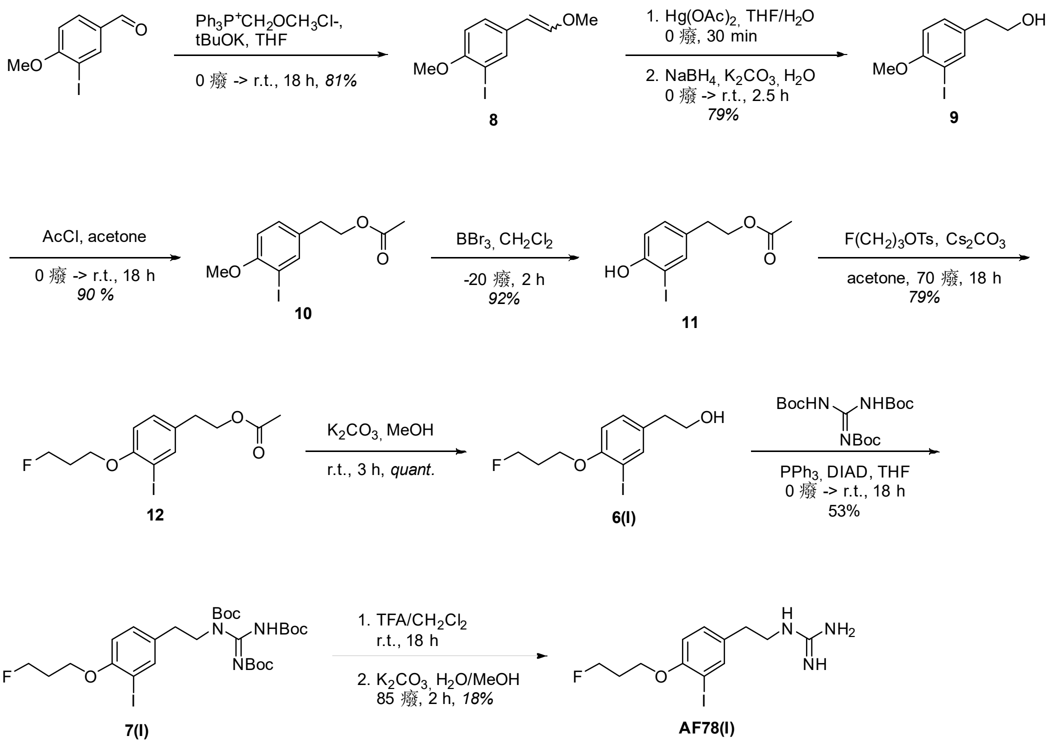

Figure S3. Synthesis of cold derivative AF78(I).

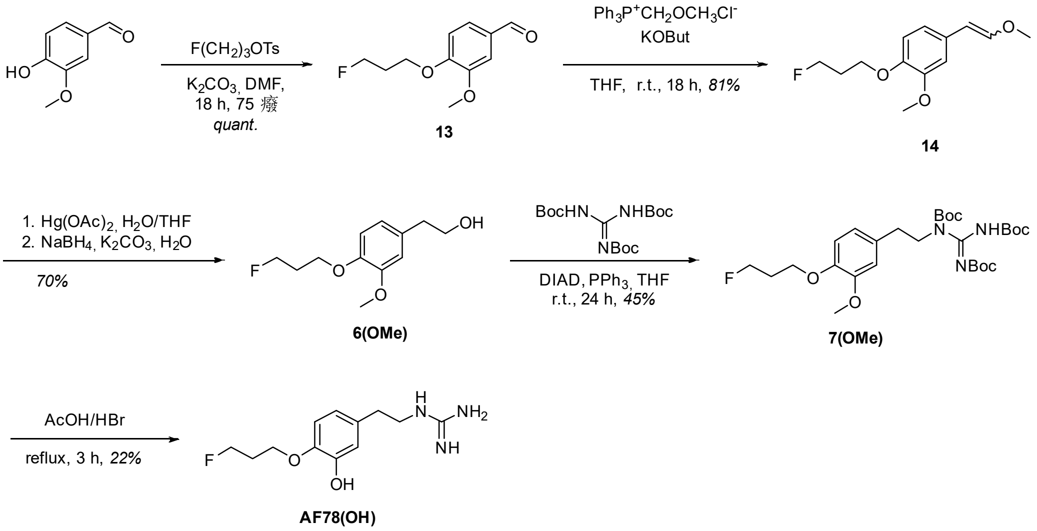

Figure S4. Synthesis of cold derivative AF78(OH).

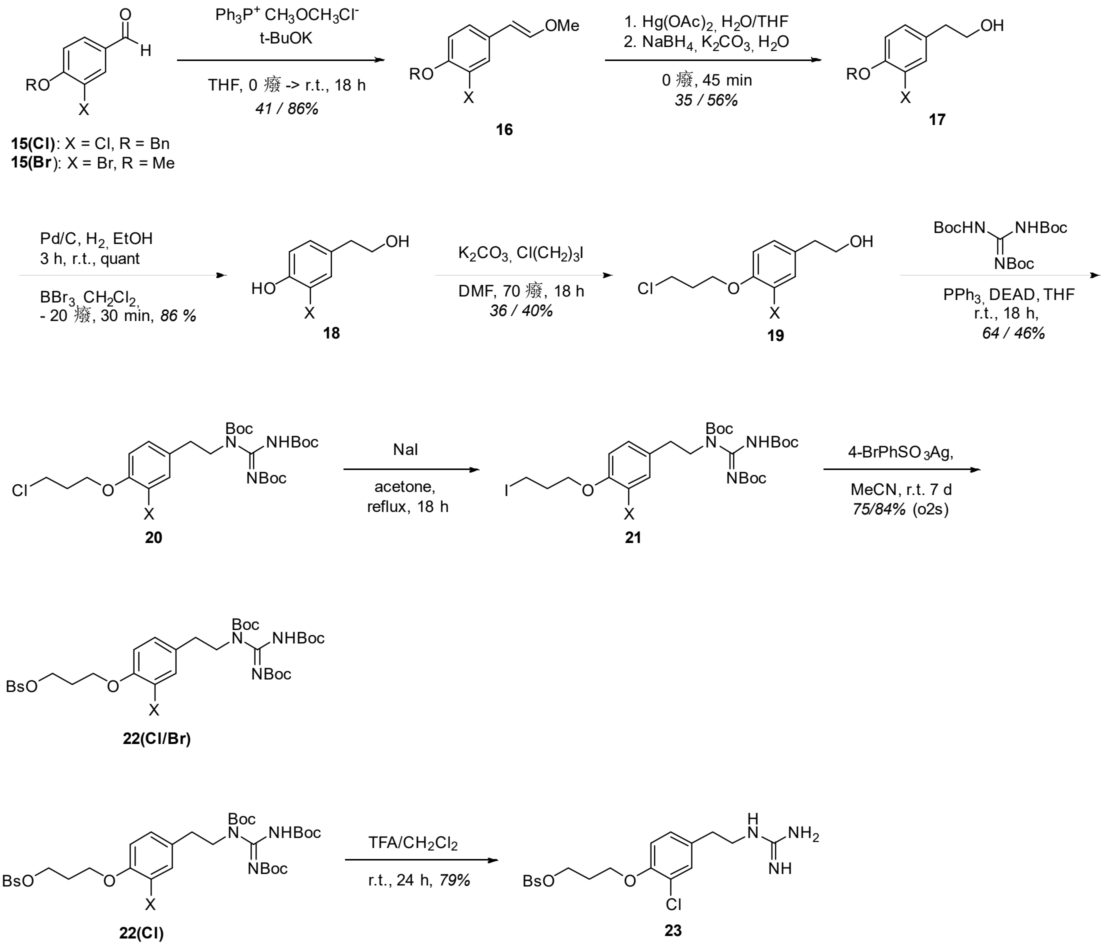

Figure S5. Synthesis of labelling precursors for [^{18}F]AF78(Cl) & [^{18}F]AF78(Br).

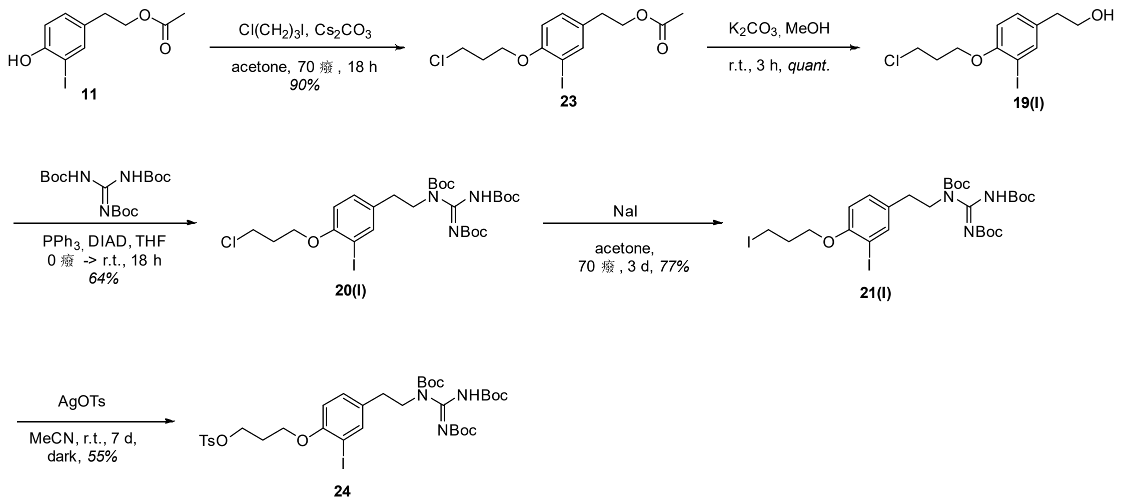

Figure S6. Synthesis of labelling precursors for [^{18}F]AF78(I).

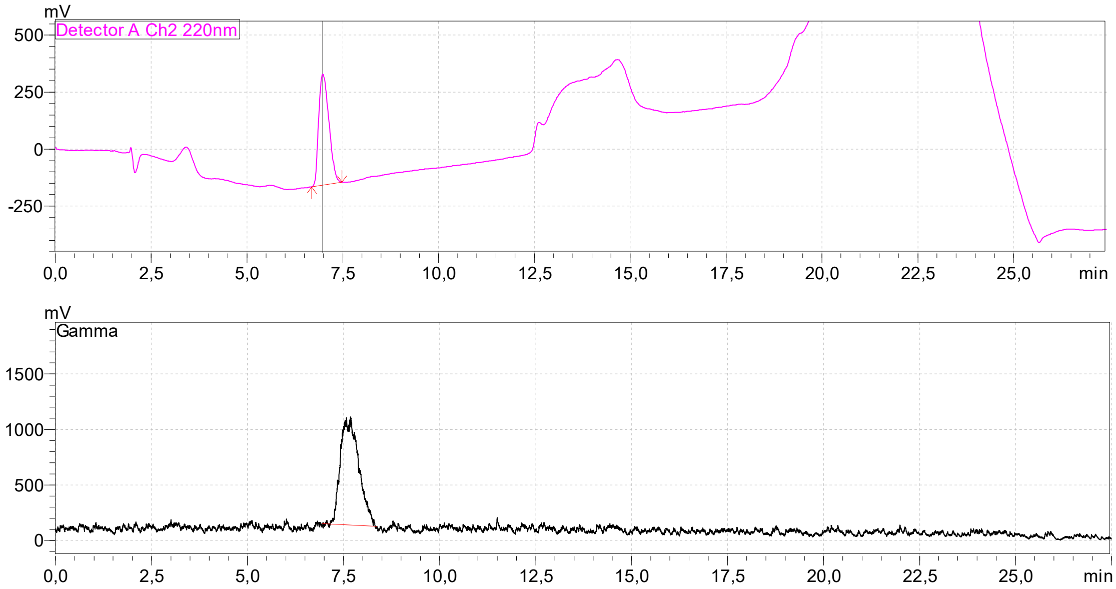

Figure S7. Analytical HPLC chromatographies of [^{18}F]AF78(Cl), UV (top, to show the retention time of cold reference) and radioactive detector (bottom, to show radiochemical purity of final product).

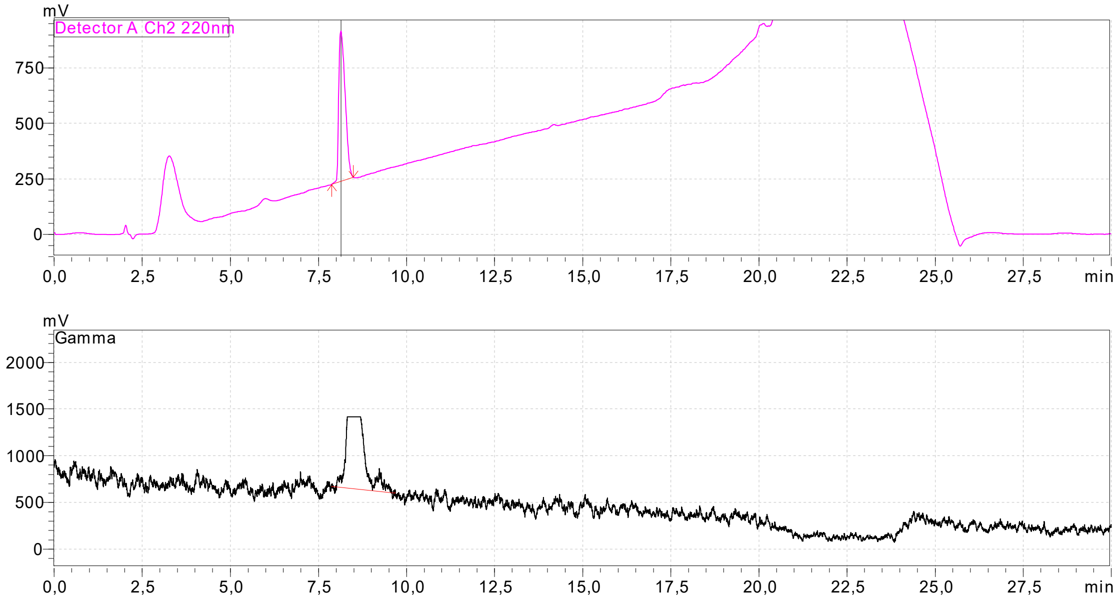

Figure S8. Analytical HPLC chromatographies of [^{18}F]AF78(Br), UV (top, to show the retention time of cold reference) and radioactive detector (bottom, to show radiochemical purity of final product).

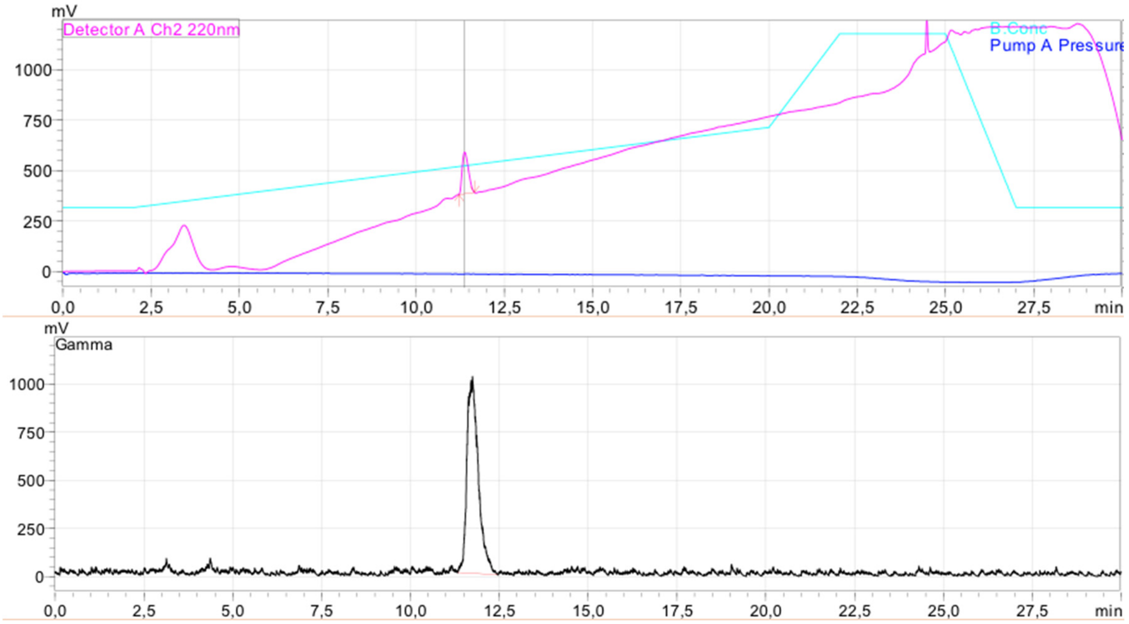

Figure S9. Analytical HPLC chromatographies of [^{18}F]AF78(I), UV (top, to show the retention time of cold reference) and radioactive detector (bottom, to show radiochemical purity of final product).

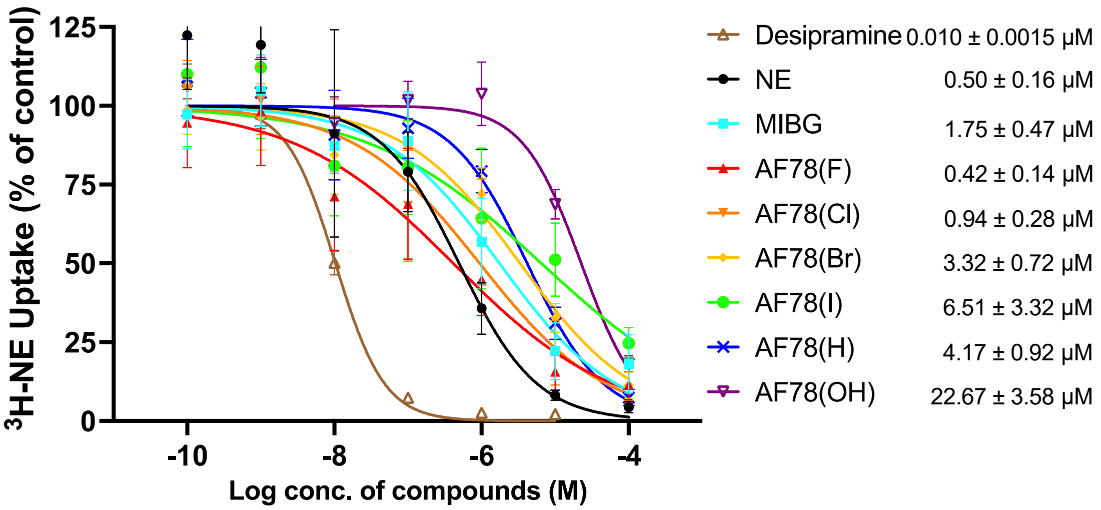

Figure S10. Dose response curves of [^{3}H]NE uptake in SK-N-SH cells in the presence of increasing concentrations of nonradioactive AF78 derivatives. Values are presented as mean ± SD for individual assays (n = 4). Each individual assay uses quadruplicate determinations of uptake at each concentration. Desipramine is used as a control [3].

Table S1. Overview of the different precursor structures and the conditions applied for labeling AF78(Cl).

**Structure of precursor**	**Quantity of precursor**	**Elution base and labeling condition**	**RCY#**	**Total time**
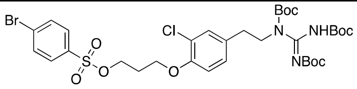	1.0 mg	1) Et_4NHCO_3 or Bu_4NHCO_3, (5 eq.), MeCN, 90 °C, 10–15 min 2) 4-6 M HCl	0.5 ± 0.1 % (n = 3)	120 min
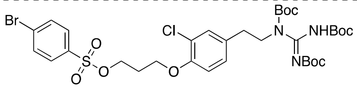	1.6 mg	1) Et_4NHCO_3 (3 eq.), MeCN, 90 °C, 15 min 2) 4 M HCl	2.6 ± 1.2 % (n = 3)	120 min
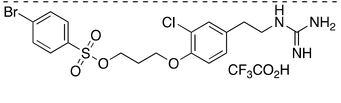	1.0 mg	Bu_4NHCO_3 (5 eq.), MeCN, 110 °C, 15 min	10.0 ± 1.0 % (n = 3)	90 min

#Radiochemical yield (RCY) is calculated from the isolated final product after HPLC and cartridge purification deducted by radioactivity loaded onto the QMA cartridge with decay correction.

Reference

1. Axelrod J, Laroche MJ. Inhibitor of O-methylation of epinephrine and norepinephrine in vitro and in vivo. Science. 1959;130:800. https://doi.org/10.1126/science.130.3378.800.

2. Mandela P, Chandley M, Xu YY, Zhu MY, Ordway GA. Reserpine-induced reduction in norepinephrine transporter function requires catecholamine storage vesicles. Neurochem Int. 2010;56:760–7. https://doi.org/10.1016/j.neuint.2010.02.011.

3. Decker AM, Blough BE. Development of norepinephrine transporter reuptake inhibition assays using SK-N-BE(2)C cells. Heliyon. 2018;4:e00633. https://doi.org/10.1016/j.heliyon.2018.e00633.
